# Learning Health Systems and Substance Use Care Cascade Achievement Among Justice-Involved Youth: A Cluster-Randomized Stepped-Wedge Clinical Trial

**DOI:** 10.1001/jamanetworkopen.2025.58222

**Published:** 2026-02-10

**Authors:** Matthew C. Aalsma, Katherine Schwartz, Dayu Sun, Lauren M. O’Reilly, Steven A. Brown, Patrick O. Monahan, Lisa Saldana, Sarah E. Wiehe, Tamika C. B. Zapolski, Leslie A. Hulvershorn, Zachary W. Adams, Allyson L. Dir

**Affiliations:** 1 Department of Pediatrics, Indiana University School of Medicine, Indianapolis; 2 Department of Biostatistics and Health Data Science, Indiana University School of Medicine, Indianapolis; 3 Chestnut Health Systems–Lighthouse Institute, Eugene, Oregon; 4 Department of Psychiatry, Indiana University School of Medicine, Indianapolis

## Abstract

**Importance:**

Adolescents involved in the youth legal system (YLS) rarely use community-based behavioral health services, despite their disproportionate risk for substance use and substance use disorders (SU/D). A care cascade framework quantifies deficits in the process by which 2 systems—YLS and behavioral health care—ensure that youths engage in indicated treatment.

**Objective:**

To test effectiveness of a cross-system learning health systems (LHS) intervention on S/UD care cascade outcomes among YLS-involved youth.

**Design, Setting, and Participants:**

This cluster-randomized stepped-wedge clinical trial was designed to improve use of SU/D treatment by youths. Eight counties in a single Midwest state were randomly assigned to 1 of 3 cohorts stepped in from preintervention control to intervention in 9-month intervals. Each county-level LHS team included juvenile probation department and community mental health center representatives. Administrative YLS records were collected from all youths aged 11 to 17 years arrested in participating counties from January 1, 2019, through March 31, 2025, and linked to Medicaid claims data.

**Interventions:**

LHS interventions were based on principles of continuous quality improvement and data-driven decision-making. Here, LHS principles were innovatively applied among collaborative teams to identify and resolve local gaps in SU/D care cascade achievement.

**Main Outcomes and Measures:**

The main outcome was timeliness of care cascade step achievement after arrest among YLS-involved adolescents. Cascade steps included screening for SU/D risk, identification with need for services, referral for services, initiation of services, and engagement in services.

**Results:**

Cascade step achievement was recorded for 5731 youths with linked YLS and Medicaid records; participants included 3538 males (62%) with a mean (SD) age of 15.4 (1.7) years at the time of arrest. A total of 1010 youths (18%) were Black, 614 (11%) were Hispanic, and 4362 (76%) were White. The LHS was associated with a significant reduction in the number of days between youth first arrest and risk screening (hazard ratio, 1.67; 95% CI, 1.12-2.23); significant interactions showed reductions in time from arrest to service initiation and service engagement for youths arrested later in the study (ie, 3.5-4.0 years after study start through end of study).

**Conclusions and Relevance:**

This cluster-randomized stepped-wedge clinical trial demonstrated that the LHS improved the timeliness of SU/D care cascade achievement among YLS-involved youth.

**Trial Registration:**

ClinicalTrials.gov Identifier: NCT04499079

## Key Points

**Question:**

What is the effect of a learning health systems (LHS) intervention, including the youth legal system and behavioral health care, on substance use or substance use disorder (SU/D) care cascade outcomes among adolescents involved in the youth legal system?

**Findings:**

In this cluster-randomized stepped-wedge clinical trial of 5731 youths, a time-to-event analysis indicated the LHS was associated with significantly reduced time between youth arrest and SU/D risk screening. Significant interactions showed reduced times from arrest to treatment initiation and engagement for youths arrested 3.5 to 4.0 years after study start (ie, post COVID-19).

**Meaning:**

These findings suggest that cross-system LHS may improve timeliness of SU/D risk screening, treatment initiation, and engagement for justice-involved youth.

## Teaser

This cluster-randomized clinical trial examines the impact of a learning health systems intervention on behavioral health care cascade outcomes using administrative records of adolescents involved in the youth legal system.

## Introduction

Overdose fatalities among US youths doubled from 2019 to 2021.^1^ Compared with peers, adolescents involved in the youth legal system (YLS) are at greater risk for substance use and substance use disorders (SU/D; 62% of youths in YLS reported SU/D^2^ vs 8% of youths not in YLS^3^). Nearly 12% of positive drug screen results from youths on probation indicate opioid and/or stimulant use. Youths in YLS also have high rates of death by overdose.^4^ However, YLS-involved youths with SU/D are rarely connected to or engaged in indicated treatment.^5^ In a meta-analysis of 22 472 youth released from detention,^6^ only 21% used SU/D services in the community. Findings from Juvenile Justice–Translational Research on Interventions for Adolescents in the Legal System (JJ-TRIALS),^7^ a multistate study of youths undergoing community supervision, indicate fewer than 10% of youth in need of behavioral health care initiated treatment.^8^ Timing of behavioral health care is also important; for example, among adults, the 2 weeks after release from prison is a peak time for overdose death.^9^ The importance of timely treatment for adolescents is further reflected in standard process measures of SU/D treatment.^10^

A care cascade framework has been used to depict and quantify deficits in the care connection and utilization process as adolescents navigate between the YLS and the behavioral health care system.^11^ SU/D care cascade steps include (1) YLS conducting SU/D risk screening, (2) identifying a need for SU/D services, and (3) referring youth to indicated services in the community. Additional cascade steps—treatment initiation (step 4) and treatment engagement (step 5)—are achieved within the behavioral health care system. Low rates of cascade step achievement, and especially long average times between arrest and subsequent step achievement, suggest the source of potential process shortfalls and points of intervention. For example, low rates of treatment initiation, or an above-average time between treatment referral and initiation, might suggest access barriers (eg, long waiting lists). Some gaps in care connection have been attributed to insufficient communication and poor relationships between YLS and behavioral health treatment agencies,^12^ which differ significantly in missions, culture, and responsibilities.^8^ At the same time, both systems play a role in the care connection process for YLS-involved youths, impacting youth outcomes.

The Alliances to Disseminate Addiction Prevention and Treatment project (ADAPT) sought to convene YLS and behavioral health care system representatives and charge them with identifying and addressing gaps in the SU/D care cascade in a novel way. The study used a learning health system (LHS) intervention. LHSs drive system-level change by engaging stakeholders in continuous quality improvement cycles.^13^ Performance data review is used to identify problems and generate solutions, which are then implemented and tested. In sum, an LHS intervenes through constant refinement of system practices based on continuous feedback grounded in clinical data.^14^ The LHS approach has proliferated across health care settings, with most success achieved within single health care systems.^15^ Although not explicitly an LHS, JJ-TRIALS incorporated LHS features of data review and continuous quality improvement; this yielded moderate success in improving behavioral health treatment engagement. Like other LHSs, JJ-TRIALS intervened only in 1 system (ie, YLS) and assessed youth outcomes based solely on YLS records.^16^ In contrast, ADAPT aimed to bring LHS into the community to convene county-level teams from both the YLS and the behavioral health care system. The LHS was designed to facilitate cross-system collaboration and improve youth outcomes by providing participants a more comprehensive picture of behavioral health care connection among YLS-involved youth.^17^ Administrative data were collected from both systems and record linked to capture cascade step achievement without altering local record-keeping practices. We aimed to examine the impact of LHS on care cascade outcomes using these administrative records.

## Methods

**Figure 1.**
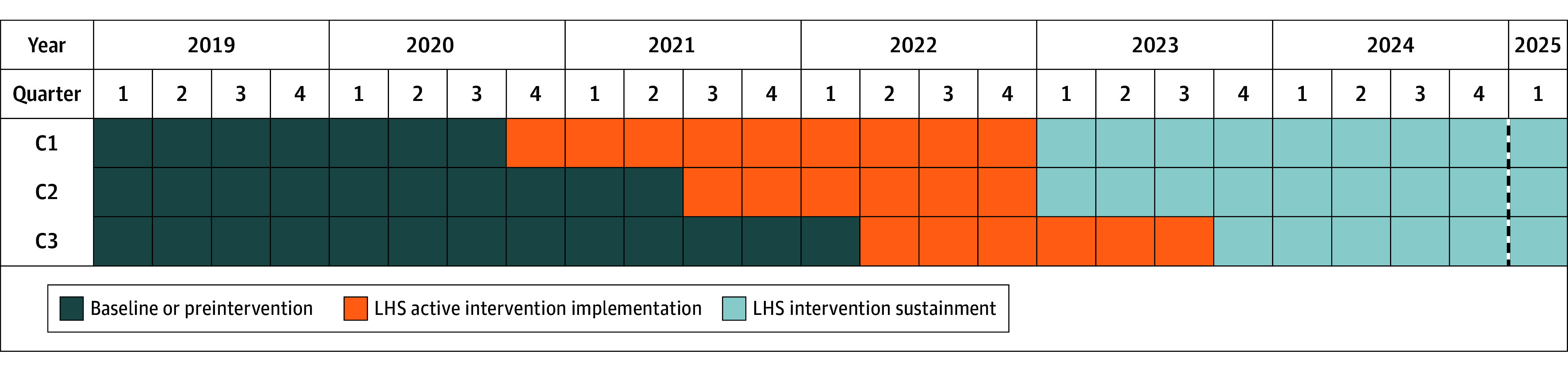
Study Timeline Depicting Stepped-Wedge Design The dashed line corresponds to the close of the Medicaid claims data available for analysis. C indicates sample cohort; LHS, learning health systems.

ADAPT was a cluster-randomized stepped-wedge, hybrid type II trial. Eight counties (ie, YLS jurisdictions) in 1 Midwest state were randomly assigned to 1 of 3 cohorts (2 counties in cohort 1 and 3 counties each in cohorts 2 and 3) using a random number generator and stepped into the study’s active 18-month LHS intervention phase with 9 months between each cohort’s start date (Figure 1). Each LHS team consisted of representatives from the YLS (ie, the juvenile probation department) and a local community mental health center (CMHC); LHS teams were charged with improving SU/D risk identification, service referral, and behavioral health care utilization for YLS-involved youths. At intervention phase start, teams were trained in LHS principles, such as conducting continuous quality improvement cycles, evidence-based treatment for adolescent SU/D, best practices in screening for SU/D risk, and the SU/D care cascade framework.^11^ The intervention entailed quarterly LHS team meetings to review data dashboards depicting county-level SU/D care cascade achievement^18^ and monthly meetings to generate and test tailored, locally relevant solutions to gaps in cascade step achievement identified during dashboard review. Examples of LHS solutions implemented within counties included (1) incorporating standardized SU/D risk screening measures into YLS intake, (2) CMHCs reserving intake appointments for YLS-referred youths to address long waiting lists, (3) applying for grants to fund CMHC services for youths with mild to moderate SU/D, and (4) training CMHC staff in evidence-based SU/D treatment modalities. Additional details about the study design and rationale can be found in the published protocol.^19^

ADAPT procedures—including a waiver of youth and guardian informed consent and Health Insurance Portability and Accountability Act authorization—were approved by the institutional review board of Indiana University. The institutional review board granted the waiver of informed consent because the research posed minimal risk to participants; data reviewed for the study were collected or generated as part of routine record-keeping practices within the juvenile justice and behavioral health care systems. The study was preregistered as a clinical trial, and results reported adhere to Consolidated Standards of Reporting Trials (CONSORT) guidelines. The trial protocol is found in Supplement 1.

### Data Sources and Record Linkage

To evaluate youth-level care cascade outcomes, administrative YLS and behavioral health care system (ie, Medicaid billing) records were collected regarding youths arrested or otherwise referred to the YLS in ADAPT counties from January 1, 2019, through March 31, 2025. Each county’s juvenile court provided local YLS records; the State Supreme Court approved data use for research purposes. The State Office of Medicaid Policy and Planning granted access to Medicaid enrollment and billing records.

Youth legal records were linked to Medicaid data at an individual level, matching records belonging to a single youth via identifiers (eg, name, date of birth, Social Security Number, residential address). The linkage was performed with Stata, release 17,^20^ using 20 deterministic matching algorithms and with RecMatch, version 1.2.2,^21^ using 5 probabilistic matching algorithms, each with conservative matching score thresholds, to determine true matches. All true matches identified through deterministic linkage were removed before the probabilistic linkage process. For each probabilistic algorithm, a manual review of potential matches under the threshold score was performed to identify remaining true matches. A final round of daisy-chaining true matches was performed to establish a unique study identification number per youth.

### Measures

#### Youth Demographic and YLS Case Information

Youth sex, race, and ethnicity were gathered from YLS database records as entered; youth age was calculated at the time of a youth’s first arrest during the study period based on youth date of birth as entered. Race (Black or African American, White, or other [including American Indian or Alaska Native, Asian, Native Hawaiian or Other Pacific Islander, multiracial, other, or unknown) and ethnicity (Hispanic or Latine or non-Hispanic or non-Latine) were included because members of racial and ethnic minority groups are disproportionally involved in legal settings. Race was collapsed into White and Hispanic compared with Black or African American or other non-Hispanic youths due to the relatively small number who identified as Black or African American and other races. Other YLS case characteristics gathered pertain to a youth’s first arrest during the study period, including most severe alleged charge and whether the charge was related to substance use, possession, or distribution.

#### Care Cascade Achievement

YLS records captured whether youths were screened for SU/D treatment need, identified as needing treatment, and referred to services. Medicaid billing data captured whether youths initiated and engaged in treatment. Cascade steps were achieved (yes or no) if completed after a youth’s first arrest during the study period, regardless of step order, and regardless of whether the step was achieved more than once per youth. By definition, a positive screen result could not occur without a youth being screened, and treatment engagement could not occur without prior initiation.

##### Screening and Identification (Steps 1 and 2)

Youths were considered screened for SU/D risk if they completed (1) an evidence-based self-report SU/D risk screener such as the CRAFFT (Car, Relax, Alone, Forget, Friends, Trouble)^22^ or the Texas Christian University drug screen^23^ or (2) an oral or urine drug test. Youths were considered identified as needing behavioral health services if they screened positive on one of the screening measures.

##### Referral (Step 3)

Youths were considered referred to services if YLS staff recorded a requirement to attend community-based behavioral health services. Requirement type values (eg, fee vs school attendance) were first coded independently by 2 investigators (K.S. and L.M.O.); coders met to resolve all coding discrepancies. Requirements could reflect court-ordered conditions of probation or court- and/or prosecutor-approved conditions of an “informal adjustment” (ie, contract between the state and youth’s legal guardian).

##### Initiation and Engagement (Steps 4 and 5)

Youths were considered to have initiated treatment if Medicaid claims indicated any use of outpatient behavioral health services following first arrest. Details regarding the coding of qualifying services have been outlined elsewhere^24^ but included a variety (eg, intake assessments, individual and/or group therapy). Youths were considered to have engaged in behavioral health services if they utilized a second qualifying behavioral health service within 6 weeks of treatment initiation per Medicaid claims.

### Statistical Analysis

Rates of cascade step achievement and time between arrest and cascade step achievement were first compared descriptively for preintervention (control) vs LHS intervention implementation phases (eTable 1 in Supplement 2). We used recommended procedures for analyzing data from a cluster-randomized stepped-wedge trial; importantly, our mixed-effects model incorporated a calendar-time fixed effect (study start to arrest date) to control for potential temporal trends and a county random effect.^25^ Specifically, using the survival package (version 3.6-4) in R, version 4.4.1 (R Project for Statistical Computing), a time-to-event analysis was conducted based on a Cox proportional hazards model for the screened, referred, initiated, and engaged cascade steps. The primary outcome was time in days from first arrest to each cascade step. To follow the expected cascade structure for this time-to-event analysis, each cascade step was defined as the number of days to reach the cascade step of interest and all higher cascade steps, whichever came first, regardless of whether the step of interest occurred. For example, if a youth was not screened for SU/D risk but was referred to behavioral health services 10 days after arrest, the time between being arrested and screened was recorded as 10 days. Similarly, if a youth was screened 20 days after arrest but was referred to services 10 days after arrest (ie, steps occurred outside of cascade order), the time between being arrested and screened was adjusted to 10 days. When the step of interest or other later steps did not occur, the censoring time was the last date of youth’s available Medicaid data or end date of available data (January 31, 2025) for youths not enrolled in Medicaid. The intervention indicator was assigned based on youth study phase at time of first arrest, attributing them into either the preintervention control phase or the LHS implementation phase (Figure 1). Each cascade step was modeled in a separate Cox proportional hazards model with intervention indicator, youth demographic covariates (ie, sex [male compared with female], race [White and Hispanic compared with Black or other non-Hispanic], and age at first arrest [continuous]) as fixed effects, and county as a random effect accounting for within-county correlation. Arrest time was included to adjust for any confounding effect of temporal or secular trends; arrest time was defined as the calendar time from trial start (January 1, 2019) to first arrest. We also tested the interaction between arrest time and treatment indicator to determine whether treatment effects changed over time. We applied the Benjamini-Hochberg adjustment to control the false discovery rate across 24 prespecified comparisons. Benjamini-Hochberg adjustment was selected over the conservative Bonferroni correction (see eTable 3 in Supplement 2) because the Benjamini-Hochberg adjustment provides greater power while maintaining rigorous control over the expected proportion of false discoveries, particularly when analyzing correlated outcomes.^26^ Two-sided *P* < .05 indicated statistical significance.

## Results

**Figure 2.**
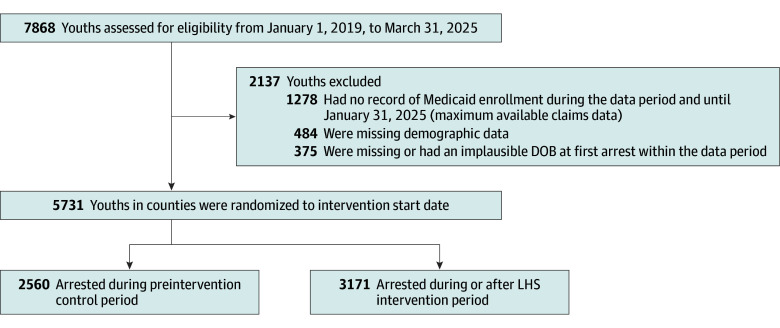
Alliances to Disseminate Addiction Prevention and Treatment (ADAPT) Study Flow Diagram DOB indicates date of birth; LHS, learning health systems.

A total of 7868 youths (aged 11-17 years) were arrested or otherwise referred to the YLS in ADAPT counties during the study period; 7009 (89.1%) were linked to Medicaid records. Of record-linked youths, 1278 with no record of Medicaid enrollment during the study period were excluded, resulting in a final sample of 5731 youths included in the analysis (2560 arrested before the intervention; 3171 arrested during or after LHS implementation) (Table 1 and Figure 2). Of the 5731 youths, 2193 (38%) were female and 3538 (62%) were male, with a mean (SD) age of 15.4 (1.7) years at the time of arrest. A total of 1010 youths (18%) were Black, 614 (11%) were Hispanic, 4362 (76%) were White, and 359 (6%) were other race.

**Table 1.** Youth Demographic and Legal System Case Characteristics by Study Phase^a^

Characteristic	Preimplementation (n = 2560)	Implementation (n = 3171)
Age at first arrest, y		
Mean (SD)	15.5 (1.6)	15.3 (1.7)
Median (IQR) [range]	15.7 (14.3-16.9) [11.0-18.0]	15.5 (14.2-16.7) [11.0-18.0]
Sex, No. (%)		
Male	1596 (62)	1942 (61)
Female	964 (38)	1229 (39)
Race, No. (%)		
Black or African American	389 (15)	621 (20)
White	1999 (78)	2363 (75)
Other^b^	172 (7)	187 (6)
Ethnicity, No. (%)		
Hispanic or Latine	256 (10)	358 (11)
Not Hispanic or Latine	2117 (83)	2606 (82)
Missing	187 (7)	207 (7)
Most severe alleged offense type at first arrest, No. (%)		
Misdemeanor	1222 (48)	1554 (49)
Felony	589 (23)	713 (22)
Substance use-related (felony or misdemeanor)	479 (19)	566 (8)

^a^ Due to rounding, percentages may not sum to 100%.

^b^ Includes American Indiana or Alaska Native, Asian, Native Hawaiian or Other Pacific Islander, multiracial, other, and unknown.

### Rates of Cascade Step Achievement and Number of Days Between Arrest and Cascade Step Achievement

**Figure 3.**
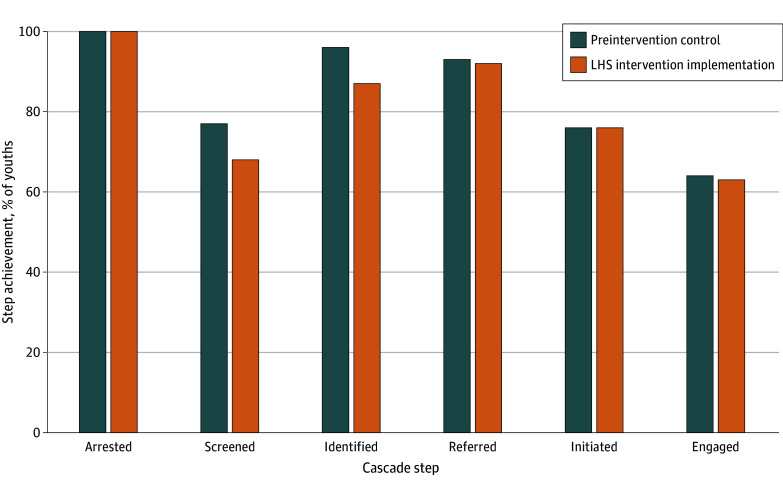
Cascade Step Achievement by Study Phase Rates were relative to previous step. Step achievement was imputed for individual youths who achieved any subsequent step. LHS indicates learning health systems.

Compared with youths arrested during the preintervention control period, a smaller share of youths arrested during or after the intervention phase achieved any cascade step (Figure 3). When comparing number of days between first arrest and any cascade step achieved, descriptive findings indicated shorter duration for youths with first arrest during or after LHS implementation (eTable 1 in Supplement 2).

### Time to Cascade Step Achievement

Analysis results, including estimated hazard ratios (HRs) of treatment indicators and covariates along with their 95% CIs, are reported in Table 2 for time to screened, referred, initiated, and engaged steps. In Table 2, the treatment effect (ie, LHS intervention vs preintervention control) was associated with change in time between arrested and screened (HR, 1.67; 95% CI, 1.12-2.23), initiated (HR, 0.68; 95% CI, 0.53-0.84), and engaged (HR, 0.64; 95% CI, 0.49-0.79). Compared with preintervention, LHS was associated with more than 1.5 times fewer days between arrested and screened. Arrest time (ie, calendar time from study start to first arrest) was not associated with time between arrest and any cascade step. However, the interaction of treatment effect and arrest time was significant for the transitions from arrest to initiation (HR, 1.10; 95% CI, 1.01-1.20) and engagement in treatment (HR, 1.13; 95% CI, 1.04-1.23), implying arrest time moderates the effect of the intervention on treatment initiation and engagement. Further exploration of these interaction results indicated that, compared with preintervention, the LHS was also associated with reduced duration between arrest and treatment initiation and engagement, but only during the later part of the study; the LHS effect began approximately 3.5 to 4.0 years after the start of the observation period through the end of the study, which roughly coincides with the COVID-19 pandemic subsiding (ie, 2022-2023) (eTable 2 in Supplement 2). For other covariates, youths who were White, female, and younger at first arrest were each significantly more likely to experience shorter duration between arrest and each of the 4 cascade steps. One exception was that youth sex was not associated with duration between arrest and screening.

**Table 2.** Summary of Time to Event Analysis Results With Benjamini-Hochberg Adjustment

Covariate	Time from first arrest to cascade step, HR (95% CI)			
	Screened	Referred	Initiated	Engaged
Treatment (LHS implementation)	1.67 (1.12-2.23)^a^	1.47 (0.65-2.30)	0.68 (0.53-0.84)^a^	0.64 (0.49-0.79)^a^
Arrest time	0.99 (0.88-1.12)	0.96 (0.86-1.07)	1.02 (0.92-1.11)	0.95 (0.83-1.07)
Treatment × arrest time	0.91 (0.74-1.08)	0.92 (0.89-0.95)	1.10 (1.00-1.20)^b^	1.13 (1.04-1.23)^a^
Male compared with female	0.96 (0.87-1.05)	0.87 (0.77-0.98)^c^	0.83 (0.74-0.91)^a^	0.81 (0.74-0.87)^a^
White and Hispanic compared with Black and other non-Hispanic race	1.21 (1.08-1.34)^a^	1.50 (1.31-1.69)^a^	1.56 (1.26-1.85)^a^	1.48 (1.13-1.82)^a^
Age at first arrest	0.94 (0.91-0.97)^a^	0.90 (0.87-0.93)^a^	0.86 (0.83-0.89)^a^	0.85 (0.82-0.88)^a^

Abbreviation: LHS, learning health systems.

^a^ *P* < .01.

^b^ *P* < .10.

^c^ *P* < .05.

## Discussion

ADAPT is the first study, to our knowledge, to test the effectiveness of implementing an LHS intervention between 2 systems, the YLS and the behavioral health care system. By using the SU/D care cascade as a guiding framework, LHS teams aimed to improve rates and timeliness of treatment need identification, service referral, and use of services among youths involved in the YLS. Findings demonstrate the utility of an LHS as an effective cross-system intervention to address system-level gaps in a care connection process requiring efforts from these 2 unique systems.

The LHS demonstrated a significant reduction in the mean number of days between arrest and screening for treatment need, as well as significantly reduced time between arrest and treatment initiation and engagement, but only after the COVID-19 pandemic had subsided (ie, 2022-2023). Although LHS was not associated with gains in rates of cascade step achievement (Figure 1), it appears that LHS participation improved the timeliness by which YLS considered youth need for treatment and the timeliness of youth connection to behavioral health care. Such results are promising, as prompt linkage to care is essential to good clinical care and outcomes.^27^ ADAPT findings also add to existing evidence that LHS interventions improve system-level efficiency.^15^ While detailing specific LHS team solutions is outside the scope of this report, policy and process change sufficient to improve timeliness of cascade step achievement requires coordination between YLS and behavioral health care systems, highlighting the impact of the LHS on cross-system collaboration and resulting benefit to service outcomes. Indeed, a previous report on findings regarding the impact of the LHS on improved collaboration and coordination between YLS and CMHCs that included most of the current investigators^28^ suggested that such collaboration may partly explain the improvements in timeliness of cascade step achievement, although further analysis is needed. ADAPT findings also echo those of JJ-TRIALS where YLS sites working closely with behavioral health agency consultants reduced the average time to treatment initiation among YLS-involved youth, again suggesting the importance of cross-system collaboration.^16^ Taken together, such results illustrate potential benefits of an LHS approach applied across 2 systems.

Contrary to hypotheses and previous findings from similar cross-system interventions,^8,17^ the LHS intervention was not associated with improved rates of cascade step achievement. One explanation for this could be that alliances did not (or could not) choose appropriate solutions to address local deficits in the care connection process; unaddressed workforce and service availability in the behavioral health system or ineffective YLS screening procedures could explain such findings. Similarly, some service gaps require complex system-level changes that in turn require time and funding to achieve, which the available data may not have captured adequately.^29^ Of note, findings were very likely impacted by the COVID-19 pandemic, which began within 6 months of the first LHS cohort intervention start. It is well understood that the pandemic resulted in widespread and significant system-level change, including informal instructions to limit arrests, extreme delays in court processing, transitions to virtual probation intakes, and more.^30^ While all ADAPT cohorts and YLS-involved youth experienced the pandemic during the study period, variations in the timing and extent of its impact on study participants may not be adequately accounted for in ADAPT design and analyses.

### Limitations

The study has limitations. While one of the primary innovations of the present study was in the use of administrative data to capture care cascade outcomes across 2 systems, well-documented pitfalls of using administrative records in research^31^ (eg, lack of contextual information, such as clinical detail; high rates of coding errors; and incomplete data capture) presented challenges here as well. For example, YLS data systems varied in available space to describe behavioral health service referrals made with any granularity. Other LHS interventions have also included patient or recipient perspectives on services as data to inform needed system solutions, which is missing from this study. Last, while the study shows an innovative use of LHS with the YLS and CMHC, results may not be fully generalizable given differences in YLS and community behavioral health system functioning across regions.

## Conclusions

This cluster-randomized clinical trial demonstrates that cross-system LHS interventions improve the timeliness of SU/D risk screening by YLS and likely facilitate timelier connection to treatment among YLS-involved youth. Although LHSs are increasingly common, to our knowledge, ADAPT is the first LHS application to improve efficiency in a cross-system care connection process for YLS-involved adolescents. Findings should encourage consideration of cross-system LHS approaches to address health care problems among vulnerable populations.

## Notes

Supplement 1.
Trial Protocol

Supplement 2. **eTable 1. **Mean Number of Days Between Arrest and Cascade Step Achieved by Study Phase **eTable 2. **Exploring the Effect of the LHS on Time From Arrest to Treatment Initiation and Engagement **eTable 3. **Sensitivity Analysis With Bonferroni Adjustment

Supplement 3.
Data Sharing Statement
