# Supplementary material for: Learning Health Systems and Substance Use Care Cascade Achievement Among Justice-Involved Youth: A Cluster-Randomized Stepped-Wedge Clinical Trial
Source: JAMA Netw Open. 2026 Feb 10;9(2):e2558222. doi: 10.1001/jamanetworkopen.2025.58222 (PMC12892156; doi:10.1001/jamanetworkopen.2025.58222)
Supplement: Supplement 1. — Trial Protocol [file jamanetwopen-e2558222-s001.pdf]

Alliances to Disseminate Addiction Prevention and Treatment (ADAPT): A Statewide Learning Health System to Reduce Substance Use among Justice-Involved Youth in Rural Communities

Principal Investigator: Matthew C. Aalsma, PhD
Funded by: National Institute on Drug Abuse
Grant number: UG1DA050070

Synopsis

Indiana, a rural Midwest state, ranks 14th nationally in drug overdose deaths. From 2016 to 2017, Hoosiers experienced the 3rd largest increase (22.5%) in overdose deaths, evidencing continuing need for intervention to reduce drug-related fatalities. Youth involved in the juvenile justice system (YJJ) bear a disproportionate burden of the addiction crisis. YJJ substance use (SU) is extremely prevalent, with a third of YJJ meeting criteria for a substance use disorder (SUD). Our research has shown that YJJ die at 1.5 times the rate of youth who have never been arrested, and a leading cause of YJJ death is drug overdose. By targeting YJJ in rural counties, our research team seeks to address the national addiction crisis at its epicenter.

Despite their high need for SU services, and the proliferation of evidence-based interventions to reduce SU, YJJ are rarely connected to needed, high-quality SU care. A cascade of care model highlights implementation gaps in YJJ achieving the full continuum of SU care (i.e., SUD risk identification, treatment referral, treatment initiation, and treatment engagement). YJJ on community supervision/probation face a unique problem accessing SU services; while the courts or probation may identify YJJ need for SU care, YJJ must receive care through healthcare agencies in the community. The primary goal of our proposed project, Alliances to Disseminate Addiction Prevention and Treatment (ADAPT) is to address this and other implementation gaps in the Cascade for YJJ. We will accomplish our goal by creating alliances between the juvenile justice system (JJ) agencies and community mental health centers (CMHCs) in eight Indiana counties.

ADAPT takes a two-pronged approach. First, we will employ a Learning Health System (LHS) to develop collaborative alliances between JJ agencies and CMHCs, organizations that traditionally operate independently. Second, we will present local Cascade data during continuous quality improvement cycles within the LHS alliances. By offering agency representatives an opportunity to view and discuss, for example, the local rate at which YJJ with SUD risk are initiating CMHC SU services, we will facilitate development of tailored, local solutions to improve the Cascade for each county’s YJJ.

Our research team is uniquely suited to implement and test the LHS alliances. We have experience facilitating JJ-CMHC collaborations in current pilot sites. Our expertise linking administrative data across juvenile justice and health systems will allow each site to quantify and visualize local Cascade data, allowing agency leaders a novel opportunity to tailor solutions to match the needs and resources of their community. Further, our team’s rich experience training agencies in evidence-based behavioral health screening and intervention ensures that local solutions to improve the Cascade will align with cutting-edge research findings, best practices, and professional guidelines. To maximize long-term sustainability of ADAPT’s JJ-CMHC alliances, we will conduct this research in collaboration with leaders from an existing statewide initiative, the Juvenile Detention Alternatives Initiative (JDAI). JDAI is a juvenile justice reform effort that utilizes data-driven decision-making and is implemented in almost 300 counties across the US. If this project is successful, the JDAI infrastructure and support for this research will inform sustainment and expansion across Indiana and the nation.

We hypothesize that ADAPT – novel LHS alliances using Cascade data to implement localized solutions to YJJ receiving evidence-based addictions care – will positively impact SU and recidivism outcomes over time. We seek to complete the following specific aims:

AIM 1: ***Implement LHS alliances between JJ agencies and CMHCs.*** We will establish LHS alliances: novel, collaborative partnerships between JJ agencies and CMHCs.

AIM 2*:* ***Generate and track local solutions to address gaps in the Cascade for YJJ in rural Indiana counties.*** Quantifying local Cascade data will enable JJ agencies and CMHCs to suggest and implement tailored, evidenced-based interventions, which will be tracked through LHS quality improvement cycles.

AIM 3: ***Assess implementation outcomes and processes.*** We will assess implementation outcomes, such as system alliance, among JJ and CHMC personnel using mixed methods.

AIM 4: ***Assess the impact of ADAPT.*** Conduct a stepped wedge cluster randomized controlled trial to assess the impact of LHS alliances on the Cascade for YJJ. We will analyze administrative data linked across JJ and health systems to assess the long-term, community-wide effects of ADAPT on public health and safety outcomes (e.g., lower rates of SU-related outcomes and criminal recidivism).

Research Plan

1 Basic Information

Study Title

Alliances to Disseminate Addiction Prevention and Treatment (ADAPT): A Statewide Learning Health System to Reduce Substance Use among Justice-Involved Youth in Rural Communities

ClinicalTrials.gov Identifier

NCT04499079. Registered 30 July 2020.

2 Study Population Characteristics

Eligibility Criteria

We describe participant eligibility for three distinct subgroups of participants:

1. Juvenile Justice (JJ) and Community Mental Health Center (CHMC) system personnel (i.e., administrators and staff) and members of the ADAPT State Advisory Board (Board; community leaders and stakeholders); study of implementation outcomes: System personnel and Board members are eligible for study enrollment if they 1) are employed by a JJ or CMHC agency within a participating county (i.e., research performance site) during the study period OR serve on the Board; and 2) are age 18 years or older.

2. Youth involved in the juvenile justice system (YJJ), administrative records sample.

   a. YJJ, total administrative records sample: YJJ are eligible for inclusion in this sample if they are 1) arrested or otherwise referred to the juvenile justice system within a participating county (i.e., research performance site) during a 10-year period, including during 5 years before the study period and during the 5-year study period; and 2) ages 11-22 years old, inclusive, at the time of their first arrest/referral within the 10-yr period of data collection.

   b. YJJ, Learning Health System (LHS) working record sample: YJJ are eligible for inclusion in this sample if they are 1) arrested or otherwise referred to the juvenile justice system within a research performance site county that is actively receiving the LHS intervention (see timeline); and 2) ages 11-22 years old, inclusive, at the time of their first arrest/referral during the LHS intervention period (see timeline). Note that the sample of YJJ included in the LHS records sample is a subset of YJJ included in the total administrative records sample. Data related to these youth will be reviewed on a quarterly basis by the LHS alliances.

3. YJJ and Parent/Guardian Dyads; qualitative interviews:

   a. Youth (YJJ) are eligible to complete qualitative interviews if they are 1) arrested or otherwise referred to the juvenile justice system within a participating county (i.e., research performance site) during the study period; 2) ages 11-17 years old, inclusive, at the time of recruitment; 3) are returning to the community after arrest/referral and justice system intake procedures (i.e., are not detained); 4) have a positive screen for substance use disorder risk; 5) a legal guardian is identified (i.e., YJJ who are wards of the state will not be recruited); and 6) speaks and reads English.

   b. Parents/legal guardians participating in the qualitative interviews: Individuals are eligible to participate if they are 1) the parent or legal guardian of a youth eligible to participate in the qualitative interviews, 2) age 18 years or older, and 3) speaks and reads English.

Inclusion of Women, Minorities, and Children / Inclusion Across the Lifespan

Inclusion of Women and Minorities

The proposed study will include a variety of participant groups from the Indiana counties included in our study: 1) JJ and CMHC system personnel; 2) YJJ, from whom we will retrieve administrative justice and health records; and 3) parents/guardians and YJJ who will participate in qualitative interviews. No matter the participant group, we will not recruit participants based on gender or race/ethnicity.

Therefore, we anticipate that our total participant sample will reflect gender and race/ethnic distributions across the greater population from which they are sampled.

The gender and race/ethnic expected distribution of system personnel is based on the demographic characteristics of all healthcare and justice system workers in the state of Indiana. We estimate that the sample will be 64% female, 98% non-Hispanic, 2% American Indian/Alaska Native, 1% Asian, 1% Native Hawaiian or Other Pacific Islander, 13% black or African American, 78% white, and 5% of more than one race.

For YJJ, the expected distribution is based on the demographic characteristics of youth in the juvenile justice system in Indiana. We anticipate that the YJJ participants will be 33% female, 95% non-Hispanic, 2% American Indian/Alaska Native, 1% Asian, 1% Native Hawaiian or other Pacific Islander, 26% black or African American, 63% white, and 7% of more than one race.

Based on the demographic characteristics of Indiana residents, we expect that parent/guardian participants will be 50.7% female, 93% non-Hispanic, 0.4% American Indian/Alaska Native, 2.4% Asian, 0.1% Native Hawaiian or Other Pacific Islander, 9.7% black or African American, 85.4% white, and 2.1% of more than one race.

Although our hypotheses are not based on sex, race, or ethnic differences, analyses will include these demographics as covariates.

Inclusion of Children / Inclusion Across the Lifespan

Children ages 11 up to age 18 are a primary focus of the proposed study. The Indiana juvenile justice system has jurisdiction over cases in which offenses were committed by a child before the age of 18, and juvenile justice records may include information regarding youth in the system up until age 23. While there is no formal minimum age of juvenile justice system jurisdiction, cases involving children younger than age 11 are extremely rare. As a practical matter, children younger than 11 years old will be excluded from study enrollment; exclusion of younger children will contribute to meaningful assessment of study outcomes, as the proposed age range is representative of the YJJ population in Indiana. We will collect administrative health and justice data regarding sampled YJJ to assess the effectiveness of an intervention (the LHS) on the substance use services care cascade for YJJ, as well as on public health and safety outcomes.

Our sample of CMHC and JJ personnel, along with Board members, will include adult participants only (ages 18 years old and older) without any maximum age restriction. This age range is justified because it is also a requirement of CMHC and JJ agency employment or Board participation to be age 18 or older.

Expertise of the Investigative Team

Our team has extensive experience in the appropriate and ethical involvement of children and adolescents in research projects. For example, many of the ADAPT faculty members are affiliated with the Adolescent Behavioral Health Research Program and have spent their entire careers conducting research with children and adolescents. Dr. Aalsma, for instance, is a licensed psychologist and has recruited especially vulnerable youth populations, including incarcerated adolescents, in longitudinal research projects. Other ADAPT co-investigators, including Drs. Adams, Hulvershorn, Dir, and Zapolski are also licensed psychologists or board certified in child and adolescent psychiatry. Our biostatistician has expertise in behavioral science, psychometrics including methodology for testing whether scale items are biased against underrepresented groups, and extensive experience with studies enrolling vulnerable populations. Thus, our team is highly qualified to ensure the ethical treatment of this population in research.

Recruitment and Retention Plan

Collectively, the ADAPT investigative team has successfully recruited youth, parents, system personnel, and community stakeholders into research and interventions for decades. Dr. Aalsma, specifically, has recruited YJJ and their parents/guardians into longitudinal qualitative and quantitative research projects. He has also successfully administered surveys and conducted qualitative interviews

and focus groups with community stakeholders like the individuals who will be included in our ADAPT State Advisory Board. Thus, the team is well-qualified to identify, recruit, and retain sufficient sample sizes for the proposed work. We describe participant recruitment and retention plans by participant type.

JJ and CMHC System Personnel and Board – Study of Implementation Outcomes

JJ personnel and CMHC personnel will be identified through publicly available staff rosters and/or supervisors' lists of contact information from all sites/counties. Many of the Board members have already committed to participating in Board-related research activities, but other community stakeholders will be approached by the ADAPT study team based on personal connections or recommendations from other Board members. System personnel and Board members will be contacted to participate through email and through in-person meetings with research staff as the opportunity arises.

YJJ – Administrative Records

To assess the effects of the LHS intervention on the substance use services cascade for YJJ (i.e., their connection to and engagement in substance use services), as well as public health and safety outcomes, we are collecting data from electronic health and justice system records regarding YJJ for both the total administrative records sample and the subsample of youth represented in the LHS working records (i.e., those reviewed quarterly). We will not be approaching these YJJ at any point in the study, so a recruitment and retention plan is not needed for this group of study participants. YJJ are identified by virtue of recorded arrest/referral to the juvenile justice system before or during the study period. Data for these YJJ will be linked to administrative health databases to form a dataset for this study.

Youth (YJJ) and Parent/Guardian Dyads - Qualitative Interviews

All YJJ completing justice system intake procedures at any research performance site will be given a flyer by the intake office. The flyer will briefly describe the study and give contact information for research personnel. Potential participants may call our research team to inquire about the study. Otherwise, our team will be provided with a list of youth (ages 11-17) who have completed intake and screened positive for substance use disorder risk using the site's standard substance use screening measure. The intake officer will provide the research team with a list of individual YJJ and parent/guardian names and their contact information, which we will use for study recruitment purposes only. Members of the research team will call potential participants and will use scripts to review participant eligibility criteria, describe the study, and schedule a time to complete assent/consent procedures with willing potential participants.

For each component of our study, we utilize a range of strategies to encourage participant retention, including:

- Emphasizing rapport-building with study participants,
- Making participation as easy as possible (providing flexible participation schedules, web-based surveys),
- Providing compensation for participants' time and/or some bonus (e.g., meals),
- Gaining permission to use a variety of methods for contacting and following up with participants, such as email, phone, text and/or social media (when appropriate),
- Maintaining frequent and/or regular contact either through the intervention or assessment activities.

3 Study Timeline

Note: "Year 1" for ADAPT consists of only 7 months, due to late inclusion as a clinical research center in the JCOIN initiative.

Study Month 1 = October 2019

	YEAR 1		YEAR 2				YEAR 3				YEAR 4				YEAR 5				
	1-3	4-6	7-9	10-12	13-15	16-18	19-21	22-24	25-27	28-30	31-33	34-36	37-39	40-42	43-45	46-48	49-51	52-54	55-
**Project Start-Up**																			
IRB and OHRP approval																			
County random assignment to stepped-wedge LHS intervention start																			
Finalize ADAPT State Advisory Board & schedule biannual meetings																			
AI training adaptation																			
Develop MOU and protocol for data sharing across sites																			
EMPACT Solutions Tableau creation																			
Develop and pilot process for local LHS data linking																			
Discuss and implement substance use risk screening across all sites																			
**Aims 1 & 2 LHS Implementation & Tracking Solutions**																			
**Overall LHS Stepped Wedge**																			
Cohort 1																			
Cohort 2																			
Cohort 3																			
AI training w/ JJ & CMHC leaders																			
LHS Implementation																			
Monthly AI consultation meetings																			
Regular LHS alliances meetings, as decided by alliance participants																			
LHS admin data collection/review																			
Monthly AI fidelity monitoring																			
Evaluation and Sustainment																			
Local data cleaning, linking & analysis																			
**Aim 3 *Implementation Analysis***																			
System personnel and Board qualitative interviews & survey data collection																			
Ongoing SIC assessment																			
**Aim 4 *Effectiveness Analysis***																			
Admin data cleaning & analysis, 5 yrs pre-study period + 5 yrs study period																			
Qualitative interviews of YJJ/Parent dyads																			
**Overall**																			
Dissemination																			
Manuscript Preparation																			

4 Protection and Monitoring Plans

Protection of Human Subjects

Protections are discussed separately for each participant type, which differ by the extent of human subjects protections required.

All procedures for this study have been reviewed and approved by the Indiana University Institutional Review Board (IRB) as a new project.

1. Risks to Human Subjects

a. Human subjects involvement, characteristics, and design.

JJ and CMHC System Personnel and Board – Study of Implementation Outcomes

We will be studying the process of implementing the LHS intervention within the juvenile justice setting, especially given that the intervention requires significant coordination between CMHC and JJ agencies. Qualitative interviews, surveys, and focus groups of system personnel and Board members (community stakeholders) will help us learn more about implementation and sustainability of the LHS intervention. Moreover, we will learn about whether or not the agencies were able to build alliances throughout the study period. We anticipate 395 staff members and 12 Board Members, by virtue of their employment at a participating agency or participation on the Board, will be eligible to participate in surveys, qualitative interviews, and focus groups. Interviews and focus groups will be recorded.

YJJ – Administrative Records

The overarching design of the proposed research is a cluster-randomized stepped-wedge testing the effectiveness of the LHS intervention across 8 Indiana counties (i.e., clinical research performance sites). Outcomes of interest include improvements in the substance use treatment cascade (Cascade) outcomes (i.e., treatment need identification, referral to treatment, and treatment initiation and engagement). We are also interested in public health and safety outcomes, including opioid-related outcomes (e.g., overdose rates, opioid-related ED utilization) and criminal recidivism (i.e., rearrests). Administrative health and justice system data will be collected to measure outcomes for this sample. YJJ will be included in the total administrative sample if they are arrested or otherwise referred to the juvenile justice system in one of the participating 8 counties during a 10-year period: 5 years pre-study period plus the 5-year study period. We conservatively estimate that approximately 3,190 YJJ will be eligible for inclusion in the study. This is a conservative estimate for several reasons, explained in greater detail in the power section for Aim 4. Briefly, it is conservative for the following reasons. First, we anticipate 2,170 adolescents will be arrested per year from the 8 counties (6,510 over 36 months including 30 months of intervention plus 6 prior months during the control or baseline condition for the step wedge design). Second, we will use administrative data from 5 years (not just 6 months) for the baseline period in the analysis; however, to be conservative, our step wedge power calculation assumes only 6 months of prior baseline administrative data (i.e., 36 months of primary administrative measurement). Third, we conservatively assume that 30% of youth will be placed out of the home (DOC and residential placement) or will migrate out of the community, resulting in an estimated 70% of 2,170 (i.e., 1,519) adolescent arrestees per year from the 8 counties (4,557 total over 36 months of primary administrative measurement). Fourth, we estimate that 30% of the 4,557 arrests will represent repeated arrests from the same youth, yielding an estimated 3,190 unique youth (i.e., 70% of 4,557) over 36 months. All data, including multiple arrests for the same youth, will be captured in the poisson model for Aim 4 analysis to decrease selection bias, increase power, and increase external validity and generalizability; however, to be conservative, we assumed in our power analysis that only 1 arrest per youth would be available (i.e., 3,190 unique youth).

Youth (YJJ) and Parent/Guardian Dyads - Qualitative Interviews

In order to further study both implementation and effectiveness of the LHS, YJJ and their parents or legal guardians will be asked to complete qualitative interviews regarding their experiences being connected to substance use services within their community. Youth residing in participating counties who are arrested or otherwise referred to the juvenile justice system during or after the LHS intervention period are eligible for the study if they also screen positive for substance use risk on a standard screening tool administered at juvenile justice system intake. We anticipate that 200 youth-parent dyads will be included in this sample, for a total of 400 participants.

b. Study Procedures, Materials, and Potential Risks.

<u>Procedures and Research Material</u>

JJ and CMHC System Personnel and Board – Study of Implementation Outcomes

Surveys, qualitative interviews, and focus groups will be used to study implementation outcomes. Research materials generally ask about participant attitudes and knowledge of organizational readiness for change or their opinions of the success of the proposed study. Board members will be asked about

potential barriers to project implementation, including local and state policies.

YJJ – Administrative Records

Research materials include electronic administrative health and justice system records from multiple sources listed below. Identifying information, including participant name, SSN, address, MRN, etc., will be used to link available data across systems for each YJJ sampled. The linkage process uses a combination of deterministic and probabilistic algorithms to identify records of the same individual across data sources. The ADAPT research team will then de-identify linked datasets for analysis. Participants will be linked to their data only via unique study ID generated by the research team. The linkage between subject identifying information and study ID will be permanently destroyed at completion of the study. Access to files containing identifiable subject information is limited to a list of investigators maintained in the project's approved IRB protocol.

- *Justice system involvement data:* We are able to capture all sample youth interactions within juvenile justice agencies from each county. Variables will include, but are not limited to, offense type, date of arrest/referrals, criminal history, length of detention stays or incarceration, case disposition requirements and recommendations, and urine drug screen results.
- *Health data:* We will collect any CMHC records (e.g., visit data, service referrals) available for all YJJ in intervention sites. We recognize that youth may receive services in locations other than the local CMHC. Thus, we will also collect administrative health data from the following sources:
    - CMHC records. We will access visit data through the LHS intervention. We will also gather initial screening data, including mental and substance use screening measures used by CMHCs. These measures will be self- or parent-report and vary based on CMHC protocols.
    - Medicaid insurance claims. We will access all behavioral health carve-out data across the entire 10-year data collection period, including services provided by the Medicaid Rehabilitation Option and health insurance coverage for youth in foster care. Medicaid claims data include all pharmacy, outpatient, emergency department, and inpatient encounters (which can be categorized by service type: mental health, substance use, physical health, reproductive health, trauma care, etc.). We will calculate visits based on ICD-10 codes, consistent with previous work.
    - DCS. A significant payor of behavioral health services, particularly residential placement, for Indiana youth is DCS. We have recently accessed these data and will do so as part of ADAPT.
    - Department of Mental Health and Addictions. We will gather Child and Adolescent Needs (CANS) data. These are functional assessments that are administered at the first behavioral health visit for all youth in Indiana.
    - Indiana Network for Patient Care (INPC). This is a statewide repository of over 90 hospitals and their patient medical record systems, and it contains the outpatient, inpatient, and emergency department encounters, as well as laboratory data, for all major hospital systems in Indiana. INPC contains over 5 billion discrete, coded clinical data elements and over 17.2 million global patient identifiers.
    - The IU Addictions Data Commons. Opioid-related health service use will be measured consistent with our previous work with behavioral health data. We will gather Emergency Medical Service (EMS), Emergency Department (ED), and hospital overdose events using chief complaint and naloxone administration data from EMS and ICD-10 code and text for ED and hospital records.

Youth (YJJ) and Parent/Guardian Dyads - Qualitative Interviews

Youth and a parent/guardian who assent/consent to participation will be interviewed in person or over the phone and be compensated for their time. Interviews will be audio-recorded and transcribed for qualitative analysis. Several dyads per county will be interviewed beginning at least 6 months post-

implementation of the LHS intervention. The dyads will be approached to complete interviews at one point and will not be contacted for future ADAPT research participation.

<u>Potential Risks to Participants</u>

JJ and CMHC System Personnel and Board – Study of Implementation Outcomes

There is almost no risk to system personnel or Board members of participating in research surveys, focus groups, or qualitative interviews. Survey and interview questions are not particularly sensitive in nature, as they generally ask about participant attitudes and knowledge of organizational readiness for change or their opinions of the success of the proposed study.

YJJ – Administrative Records

The potential risks to the participants are minimal, and all risks of harm would be associated with loss of confidentiality. YJJ in this sample will not be contacted by the investigators. All investigators have experience in safe-guarding sensitive information and data, including individually-identifiable clinical and incarceration data. Identifiable data will be extracted only to enable the rest of the study data to be extracted electronically; names, address, and identification numbers will then be replaced by a unique study ID. Subsequently, files for analysis will contain only the study ID. The confidential key file linking the identifiable data to the study identifier will be stored in a password-protected file behind the university firewall.

Youth (YJJ) and Parent/Guardian Dyads - Qualitative Interviews

The confidential information at risk of being exposed potentially includes a youth's criminal activity, description of their social and family environment, description of their relationship with their parents, and details of their behavioral health problems and behavioral healthcare utilization, among other sensitive personal information. If information of this nature was shared outside of intended contexts (legal, clinical, research, etc.), then participants could experience psychological and social harm. For example, participants may be stigmatized and/or embarrassed in front of peers. Information revealed by a breach of confidentiality could also potentially impact the court cases the youth are involved in or their treatment within the justice system. However, these risks are not significantly greater than otherwise faced by participants when they experience the justice system or utilize behavioral health services.

2. Adequacy of Protection Against Risks

a. Informed Consent and Assent

Assent/Consent/Study Information Sheets described below will all include draft language from JCOIN regarding the transfer and use of their de-identified data across JCOIN hubs, the MAARC, and HEAL, per our Data Use Agreement with JCOIN.

JJ and CMHC System Personnel and Board – Study of Implementation Outcomes

Consent will be sought prior to any human subjects activity with each potential study participant. By careful orientation to the nature, risks, and benefits of the research, we will provide potential study participants with all information necessary to make an informed decision to consent to participate or to decline involvement in the study. Research personnel will specifically remind potential participants that their participation is voluntary, that they may decline to participate in any or all individual components of the study. At the beginning of each survey, focus group, or individual interview, participants will receive a copy of the study information sheet or consent form that outlines: (a) the voluntary nature of participation, (b) the right to withdraw from the study at any time, (c) the purpose of the study and how the data will be used, (d) the types of data to be collected, (e) an explanation of confidentiality and the exceptions to it, (f) possible risks to the participants, (g) potential benefits to the participants, and (h) contact information if they have questions or concerns regarding the assessment procedures.

YJJ – Administrative Records

We will not be obtaining informed consent from the individuals whose information is extracted from administrative databases for purposes of this research study. This study will be looking retrospectively at thousands of records. Obtaining consent for this quantity of participants could not be practically

carried out.

Youth (YJJ) and Parent/Guardian Dyads - Qualitative Interviews

We will follow the same consent procedures for parents/guardians as described for system personnel, and the same principles will be applied in the assent procedures for YJJ.

b. Protections Against Risk

In general, protection against risk to participants will be accomplished through thorough training of research staff; careful orientation of potential participants as to the nature, risk, and benefits of the research; strict adherence to study protocols; and regular surveillance for adverse events. Additionally, ADAPT will receive an NIH Certificate of Confidentiality to further protect information shared by justice-involved youth and their parents/guardians.

The following steps will be taken to protect the confidentiality of all data generated and collected throughout the course of the study:

- Data access will be limited to a specified group of programmers and researchers identified as study personnel within our IRB protocol. An audit trail will be maintained to track all user-access to the data.
- All study investigators and research team personnel will be trained in the importance of confidentiality and trained to follow all rules related to handling confidential data. All protocols will be reviewed at least monthly during team meetings.
- All data sets resulting from this study will be kept exclusively on a secure internal server behind the university's firewall, access to which requires administrative approval and password. The internal server is encrypted at the storage, backup, and sharing levels, thereby maximizing protections against breaches of confidentiality.
- The research team will not be permitted to copy the raw data or to transmit the raw data other than through a secure server connection.
- The data will not be stored in hard copy, unless the data are in aggregate format (e.g., data displayed in a table, presentation, manuscript, or output from statistical software). All hard copies will be stored in a locked filing cabinet in the PI’s office suite when they are not in use.
- Results of the study will be reported only in the aggregate and without other identifiable data, with attention to issues of statistical disclosure.

JJ and CMHC System Personnel and Board – Study of Implementation Outcomes

For system personnel and Board members, personal links to surveys will be sent via email, and the surveys can be completed from a private location, such as from an office or home computer. For any individual completing qualitative interviews, participants will be able to participate over the phone, and research assistants completing the interviews will instruct participants to respond to questions only if other individuals are out of earshot. For qualitative interviews completed in person, the research assistant will ensure the interview is conducted in a private location.

YJJ – Administrative Records

Detailed procedures for handling administrative health and justice system data follow:

- *Data extraction.* We will extract all juvenile justice encounter data and all CMHC or other outpatient health visit-level data, including laboratory data and the other demographic, socioeconomic, and clinical variables of interest. We will take all precautions to keep name, social security number, date of birth, and address data confidential at all times. Each participant will be assigned a unique study ID for tracking. The records linking the identifying data and study ID will be encrypted, password protected, and kept in a locked office. These will be maintained only during the study period for administrative purposes to allow for longitudinal tracking. Only individuals named specifically in the IRB protocol will have access to records linking identifiable information to study ID. All data management and analysis will be performed by professionals with appropriate training and skills.

We will destroy linkages between individual identifiers and study identification numbers at the conclusion study period. Thus, data will be identified only by study ID and no linkage to individual identifiers will be maintained.

- *Data linking.* After the YJJ sample is identified, data from all juvenile justice system and health records will be extracted. Standard pre-processing of individual identifiers is completed first. We create phonetic transformations using “Soundex” and “NYSIIS” algorithms that help to eliminate misspellings and other errors in names. We will randomly sample and manually review algorithm-declared matches and non-matches, iteratively refining the algorithm to desired performance (using alternative strategies as indicated such as algorithms stratified by gender or weighted by name frequency by race/ethnicity). Authorized members of the research team will link the matched records from the health and justice data repositories using the study ID. The resulting file will be stripped of all identifying data except for the study ID. All identifying data will be kept separate from the clinical and justice system data and will be encrypted, password protected, and stored behind the university firewall.
- *Destruction of identifiers.* All identifiers, including name, address, social security number, medical record number, and date of birth, will be destroyed as soon as data are appropriately linked. At the end of the study, all electronic data files will be destroyed using guidelines developed by the National Institute of Standards and Technologies Guidelines for Media Sanitization. A recommended process includes using Drive eRazer Ultra, which completely purges information collected on a hardware device. This device completely destroys data files by overwriting them in a series of passes. This application will be used to remove data from the hard drive at the completion of the study period using 99 passes. Each pass overwrites the data with zeros and will result in a hard drive that is completely clean of all data. All discarded output will be destroyed using a dedicated confetti shredder. All hard copy output that is not destroyed will be stored in the locked file cabinet when not in use by the PI. No hard copy data will leave the PI’s office in other than aggregate format such as graphs or tables that would be used for abstracts, presentations, or scientific papers. At the completion of the study period, all non-aggregate hard copy data will be shredded.

Youth (YJJ) and Parent/Guardian Dyads - Qualitative Interviews

Some additional measures of protection against risk will be provided to these participants.

Protection of privacy. All YJJ and parents/guardians will be given a choice of location to complete research interviews. For example, they may choose to complete interviews at home, which will help protect their privacy from anyone outside the research team while participating in the research. Youth and parents/guardians will be asked to complete interviews in separate rooms out of earshot of each other, such that neither person in the youth-parent dyad can here the other's interview responses. Participants will also be able to participate over the phone, and research assistants completing the interviews will instruct participants to respond to questions only if other individuals are out of earshot.

Protection against psychological discomfort. Qualitative interview questions will touch on potentially sensitive topics, including substance use behavior, treatment utilization, and parent-youth relationships. Participants will be informed of this possibility through assent/consent procedures, reminded that they may skip questions or decline answering. If a participant expresses distress directly to the research team, they will be provided the name and contact information of several licensed behavioral health providers on our research team and other local resources as needed.

c. Vulnerable Subjects

While we are not directly interacting with the majority of YJJ who are represented in the administrative databases, we are collecting juvenile justice history data for thousands of YJJ across 8 sites. Data will include information related to the course of their juvenile justice involvement including charges, referrals, and other important events with their juvenile justice file, such as case disposition, probation involvement, and any instances of detention or incarceration.

In order to access, analyze, and link this administrative data for research purposes, we are required to obtain approval from the Indiana Supreme Court. The IUSM team has successfully received approval for this data for past studies and, due to our strong support from the judiciary we expect the process to take less time than usual.

As described in the previous section, the IUSM team has strong protocols in place to protect this sensitive data.

3. Potential Benefits of the Proposed Research to Research Participants and Others

Overall, we anticipate that all YJJ in participating counties will benefit from the LHS alliances. This intervention is designed to increase youth connection, initiation, and engagement in needed substance use treatment; reduce opioid-related harms; and reduce recidivism. If these aims are met, the benefit should be felt across all counties participating. Furthermore, we hope to improve the alliance between juvenile justice system agencies and community mental health center personnel, increasing their communication and improving processes to better outcomes for youth involved in the system.

4. Importance of the Knowledge to be Gained

Our overarching goal is to learn whether the LHS (and related local solutions) effectively improve the substance use disorder care continuum and get justice-involved youth the behavioral healthcare that they need. We will also learn whether this intervention may help address the national opioid crisis by preventing adolescent substance abuse and addiction and reducing recidivism.

We will also learn more about the benefits and challenges faced by the justice system and CMHCs when attempting to implement an intervention of this nature.

Finally, we anticipate that science and society will benefit from our increased knowledge of whether the LHS alliance is sufficient to improve outcomes for youth in the juvenile justice system.

Data and Safety Monitoring Plan

1. Summary of the Protocol

The proposed study is a hybrid Type 2 implementation-effectiveness trial in which 8 counties will be cluster-randomized to a stepped-wedge start date for receiving the LHS intervention to improve the substance use care cascade for youth involved in the juvenile justice system. Study participants include juvenile justice agency and community mental health center staff, as well as justice-involved youth and their parents/guardians.

As detailed in the Protection of Human Subjects section, study participants of all types face minimal risks associated with study participation. Most risks would be associated with potential loss of confidentiality or minor psychological discomfort.

2. Roles and Responsibilities

Dr. Aalsma (PI) will be responsible for monitoring the safety of the study, executing the DSMP, and complying with reporting requirements. These efforts will be co-led by Dr. Adams. Dr. Aalsma will provide a summary of the DSMP report to NIDA each year as part of the progress report. The DSMP report will include the participants' demographic characteristics, expected versus actual recruitment rates, any quality assurance for issues that occurred during the past year, a summary of adverse events, and any actions or changes with respect to the protocol. The DSMP report to NIDA will include the results of any data analysis.

3. ADAPT Safety

ADAPT safety monitoring will be conducted by Drs. Aalsma and Adams throughout the study in compliance with the following requirements of the Indiana University IRB's continuing review process:

1) Tracking of subject accrual (enrollment, drop-outs, demographics)
2) Timely and appropriate reporting of informed consent process deficiencies, protocol deviations, privacy breaches, conflicts of interest, and/or changes in personnel

3) Ongoing monitoring and appropriate reporting of adverse event activity
    a. Internal: i) frequency of unexpected, related or possibly related, and serious or more prevalent than expected adverse events; ii) frequency of internal deaths occurring during the study or within 30 days of study termination, even if expected or unrelated
    b. External: frequency of unexpected, related or possibly related, and serious adverse events
4) Interim assessment of risk/benefit relationship in reference to adverse event occurrences, preliminary observations, and emerging information
5) Timely and appropriate IRB submission of safety-related documents such as audit reports, sponsor progress reports, and other materials or communications that might impact the safe conduct of this study
6) Active cooperation with the IRB and other applicable entities in the event of a random or for-cause internal or external audit

4. Interim Analysis and Stopping Rules

Formal interim analysis is planned for this study at 18-month point. The PI may prematurely terminate the study if deemed necessary, although this is not expected due to the minimal risks involved in conducting the study.

5. Monitoring and Reporting

1) Identification. Potential risks identified for participants will be listed in the IRB-approved informed consent document. Additional unknown risks might occur and, if so, will be identified through diligent investigator monitoring throughout the conduct of this study.
2) Detection. During the informed consent process, participants will be advised of the potential risks of participation as identified in the IRB-approved informed consent document. Participants will be advised during the informed consent process that they should promptly inform the investigators of any concerns regarding adverse events related to participation in the study. Safety parameters will be followed by the PI as outlined above.
3) Grading. Adverse events will be assessed and graded according to the IU IRB Adverse Event Reporting Policy:
    a. Expected/Anticipated Adverse Event: Identified in nature, severity, or frequency in the current protocol, informed consent, investigator brochure, or with other current risk information.
    b. Unexpected/Unanticipated Adverse Event: Not identified in nature, severity, or frequency in the current protocol, informed consent, investigator brochure, or with other current risk information.
    c. More Prevalent: Occurs more frequently than anticipated or at a higher prevalence than expected.
4) Attribution. Adverse events will be attributed to study participation according to the IU IRB Adverse Event Reporting Policy:
    a. Unrelated: There is not a reasonable possibility that the adverse event may have been caused by participation in the study.
    b. Possibly Related: The adverse event may have been caused by participation in the study; however, there is insufficient information to determine the likelihood of this possibility.
    c. Related: There is a reasonable possibility that the adverse event may have been caused by participation in the study.

   d. Seriousness: Results in death, is life threatening, requires inpatient hospitalization or prolongs existing hospitalization, results in persistent or significant disability/incapacity, or any other event that may jeopardize the participant's health and may require medical or surgical intervention to prevent one of the other outcomes listed in this definition.

5) Reporting. Adverse events experienced by participants will be reported using the IRB's password protected on-line adverse event reporting system, in keeping with the IU IRB Adverse Event Reporting Policy as follows:

   a. Internal Adverse Events. An internal adverse event is reportable if the occurrence meets all three of these conditions: 1) is unexpected, 2) is related or possibly related to participation in the research, and 3) is serious or places participants or others at greater risk of physical or psychological harm than was previously known or recognized. Internal adverse events are not reportable under these conditions: 1) expected and not more prevalent than expected, whether related or unrelated, and 2) unexpected and unrelated, regardless of seriousness, aside from death.

   b. Internal Deaths. All internal deaths occurring during the conduct of the study or 30 days post-termination from protocol are required to be reported as adverse events even if they are expected or unrelated.

   c. IRB Reporting Schedule for Adverse Events. For any unexpected and related or possibly related, serious or more prevalent event occurring during a research study, a report must be made through the IU online reporting system as soon as possible but no later than 10 working days after the investigator first learns of the event. All other (expected adverse events) are reported as aggregate data at the time of IRB continuing review.

   d. Report of Changes or Amendments to the Protocol. The annual DSMP report will describe any minor actions or changes with respect to the protocol. In the unlikely event that major changes are required, the Principal Investigator will discuss the needed changes with the NIDA Program Official first, reach a consensus agreement on the changes with the Program Official, and then provide a written description of the changes to NIDA.

6. Data and Safety Monitoring Board

NIDA will establish a Data and Safety Monitoring Board (DSMB) that will be responsible for safeguarding the interests of all Justice Community Opioid Innovation Network (JCOIN) participants. The DSMB will be comprised of external experts who will be responsible for monitoring study data relevant to bioethics and the safety of the study participants and impacts on study operations and procedures. JCOIN contact PIs will be responsible for providing the DSMB timely information about their projects as requested, including reporting to the DSMB at least annually. The DSMB will make recommendations to NIDA with respect to: 1) Participant safety, confidentiality, and informed consent, including notification of adverse events; 2) participant burden; and 3) impact of proposed ancillary studies and sub-studies on participant burden and overall achievement of the main study goals.

5 Study Team

Science Team by Aim (Investigators, Research Consultants, Community Leads)

Role	Name and Title	Aim	Expertise
PI	Matthew C. Aalsma, PhD<br>Professor of Pediatrics, IUSM	All	Alliance building among JJ and CMHCs; YJJ mental health and substance use disorder prevalence, risk screening, and treatment utilization; implementation science; mixed method data analysis; community based participatory research; multi-site research
KP	Nancy Wever<br>Director, Indiana Juvenile Detention Alternatives Initiative	All	"Justice System Senior Leader"
KP	Douglas Huntsinger<br>Special Assistant for Drug Prevention, Treatment and Enforcement, IN Office of Governor Eric J. Holcomb	All	"Community Health Senior Leader"
Co-I	Malaz Boustani, MD<br>Richard M. Fairbanks Professor, IUSM	1	LHS; Agile Implementation training and execution
Co-I	Peter J. Embi, MD<br>Professor of Medicine, Regenstrief, Inc.	1	LHS development; data storage and transfer policies (Indiana Addictions Data Commons)
Co-I	Nicolas Terry, JD<br>Professor of Law, McKinney School of Law	1	Healthcare policy analysis; addressing ethical and legal issues related to data sharing
C	Gregory A. Aarons, PhD<br>Professor of Psychiatry<br>Director, Child and Adolescent Services Research Center, UCSD	1	EPIS model of implementation
Co-I	Zachary W. Adams, PhD<br>Assistant Professor of Psychology, IUSM	2	Evidence-based behavioral health treatment for adolescents; training behavioral healthcare providers
Co-I	Leslie A. Hulvershorn, MD, MSc<br>Associate Professor of Psychiatry, IUSM<br>Medical Director, Indiana Division of Mental Health and Addiction	2	Development and trajectories of adolescent substance use disorders; Evidence-based behavioral health treatment for adolescents
Co-I	Allyson L. Dir, PhD<br>Assistant Professor of Psychiatry, IUSM	2	Evidence-based behavioral health treatment for adolescents and emerging adults
Co-I	Tamika C.B. Zapolski, PhD<br>Assistant Professor of Psychology, IUPUI	2	Substance use disorder development and trajectories among Black adolescents; Evidence-based behavioral health treatment for adolescents
Co-I	Lisa Saldana, PhD<br>Senior Research Scientist, OSLC	3	Implementation science; Stages of Implementation Completion (SIC)©
C	Cara C. Lewis, PhD<br>Associate Investigator, Kaiser Foundation Health Plan of Washington	3	Implementation outcomes measurement
Co-I	Sarah E. Wiehe, MD, MPH<br>Associate Professor of Pediatrics<br>Chair, Children's Health Services Research, IUSM	4	Linking administrative datasets using probabilistic algorithms; data analysis; community based participatory research

Co-I	Patrick Monahan, PhD<br>Professor of Biostatistics, IUSM	4	Data analysis; psychometrics
Co-I	Shaun Grannis, MD<br>Director, Center for Biomedical Informatics, Regenstrief Institute, Inc.	4	Linking administrative datasets using probabilistic algorithms; Indiana Addictions Data Commons
Co-I	Kosali Ilayperuma Simon, PhD<br>Professor of Public and Environmental Affairs, IUB	4	Health economics analysis

6 Clinical Performance Sites

Eight counties, i.e., JJ system jurisdictions, across Indiana participated in ADAPT. A local CMHC per site also participated. Names of the counties and CMHCs are withheld from publication.

LHS Alliances by Site

LHS alliance teams will be composed of existing JDAI local data teams. The local JDAI data teams consist of JJ personnel and community members. The goals of the data teams are to assess local data to determine youth risk level and to improve outcomes among YJJ. LHS alliance teams will utilize these teams, along with additional team members from the local CMHC, to conduct continuous quality improvement cycles.

State Advisory Board

The ADAPT State Advisory Board will consist of delegates from state agencies, academic consultants, local practitioners, and youth and parent stakeholders identified through local IJDAI partners. The purpose of the State Advisory Board will be to 1) evaluate the strengths and weaknesses of ADAPT; 2) recommend state and local policies to enhance impact; 3) disseminate study results, and 4) explore sustainability. The Advisory Board will receive an annual report on ADAPT progress and meet twice yearly with Dr. Aalsma. ADAPT co-Is, Professor Nicolas Terry and Dr. Kosali Simon, will convene a policy gap workgroup of board members, on an as-needed basis to conduct policy analyses relevant to ADAPT.

7 Study Design

Learning Health System

A central goal of this project is to build a collaborative alliance between JJ and CMHC agencies. The reason to do so is to improve substance use treatment referral and substance use treatment outcomes for YJJ. An LHS includes continuous quality improvement and the development of professional teams to develop interventions and evaluate the outcome. We will use already established implementation methodology (“Agile Implementation”) for the LHS intervention. Agile Implementation utilizes an 8-step process to effect change. We will adapt the AI training for the current setting. In order to make the continuous improvement cycles effective, our research team will link local juvenile justice and community mental health center data for each site. We will do so on a quarterly basis and the data can be used during local committee meetings. Our research team has extensive experience conducting deterministic and probabilistic linking and cross-sectional data linking of justice-involved youth to CMHC data of this size will be straightforward. We will also work with the data programmer affiliated with the Juvenile Detention Alternatives Initiative (JDAI), Mr. Melchi, to develop these tables for informing interventions. He has done this work at the local and state level for many years.

Substance Use Risk Screening

Based on research and our pilot data, substance use risk screening will need to be implemented universally to assess the extent of each community’s need for connection to substance use treatment services.

Assessing Outcomes

Implementation outcomes. This is a hybrid Type II effectiveness implementation trial. Thus, we are interested in the implementation of the LHS intervention. We will assess implementation with self-report surveys, qualitative interviews, and focus groups. We will also include the Stages of Implementation Completion (SIC) measure, an 8-staged observation-based assessment tool created as part of a large-scale randomized implementation trial comparing two implementation processes. The SIC stages - which we will apply throughout the study period to measure implementation outcomes - range from: Engagement with the academic team to achievement of practitioner Competency and map onto three well-accepted phases of implementation (Pre-Implementation (i.e., exploration and planning), Implementation, Sustainability). The SIC has demonstrated valid and reliable measurement of implementation activities, specifically the proportion and duration of implementation activity completion. Lastly, we will complete three sets of qualitative and focus group interviews. By utilizing a mixed method approach, will be triangulate results and gather a more nuanced picture of implementation issues.

Substance use care cascade. A primary outcome of interest is the SU care cascade (Cascade). The Cascade includes a spectrum of outcomes. At any point in the Cascade, a young person can “drop out.” The earliest stage of the Cascade occurs within the juvenile justice system: identification of treatment need (i.e., screening and assessment) and referral to services. The latter end of the Cascade occurs within the CMHC: treatment initiation, engagement, and completion. We are interested in determining whether the LHS intervention is effective in improving the Cascade. We will evaluate this outcome utilizing administrative data on the entire population of YJJ across all 8 sites. We will gather Cascade data from the local CMHCs as well as Department of Child Services (DCS) and Medicaid. The reason for gathering data from DCS and Medicaid is they both provide funds for outpatient services. Thus, if a young person receives substance use services outside of the local CMHC, we will be able to gather at least a portion of it from these data sources. We may find that the effectiveness of LHS is improved for young people seeking services outside of the CMHC. With that said, we have collaborated with the largest CMHC of each jurisdiction and most jurisdictions only have one CMHC located in their county. We will also be able to control for time spent outside of the community by controlling for commitments to Department of Corrections (gathered from the juvenile justice data set) and residential placement (gathered from DCS and Medicaid).

Criminal recidivism. We will also assess whether the LHS intervention is effective in limiting recidivism. We will utilize the existing juvenile justice information system to assess recidivism. We will focus on the prediction of new criminal charges rather than charges associated with previous criminal behavior (e.g., technical violation, probation violation and warrants).

Opioid-related outcomes. By linking with data collected through the Regenstrief Institute Addictions Data Commons, we will be able to assess a variety of outcomes. This includes hospital and Emergency Department visits for opioid use disorders (OUD) as well as emergency medical service runs due to opioid use. These data elements are now incorporated within the Addiction Data Commons.

Outcome Measures

Type	Name	Time Frame	Brief Description
Primary	Effective Collaboration (Quantitative)	Continuous	The SIC will allow for continuous measurement of implementation success. Assessment of the SIC results will be ongoing with subsequent evaluation at the completion of the intervention period.
Primary	Effective Collaboration (Qualitative and Mixed Method)	42 months (6 months pre- and 6 month post-30-month LHS intervention period)	We will use a mixed method approach in evaluating the alliance, as measured by the effective collaboration survey as well as qualitative interviews, to assess for changes over time, between-system changes, and within-system changes.
Primary	Cascade	10 years, total	We will use administrative data to compare the effectiveness of

		admin sample; 42 months LHS intervention sample	the LHS intervention on the Cascade (i.e., substance use treatment need identification, referral to services, initiation of services, and engagement in services.
Primary	Criminal recidivism	10 years, total admin sample; 42 months LHS intervention sample	We will evaluate criminal recidivism across the 10-year administrative data collection period. We will assess if Cascade improvement is associated with decreased criminal recidivism. We will also assess if there are intervention-specific effects on criminal recidivism.
Primary	Opioid-related outcomes	10 years, total admin sample; 42 months LHS intervention sample	We will use administrative data to assess opioid-related outcomes. We will assess if Cascade improvement is associated with decreased opioid-related outcomes. We will also assess if there are intervention-specific effects on opioid-related outcomes.

Statistical Design and Power

Aim 3 Implementation Outcomes Analyses

The analyses will proceed in two steps.

Step 1. We will use standard step wedge analytic methods (see citations in reference list for PASS report below). We will use linear mixed models to analyze repeatedly measured implementation variables to determine whether implementation variables change over time from the control to intervention conditions. Organizational survey data will be collected at the county level (from 164 JJ staff) and from the health system level (from 231 CMHC staff) for a total of 395 surveys collected at six time points at 6-month intervals: Study Months 8, 14, 20, 26, 32, 38. The following continuous scale scores will be analyzed in a separate model as the dependent variable: perceived readiness to implement new innovations (ORIC), climate support of adopting new innovations (ICS), organization's leadership support of implementation of evidence-based practice (ILS), and collaboration alliance between JJ/CMHC (Effective Collaboration subscale). The models will contain random effects to account for the clustering of survey participants within county and health system organizational levels. The models will contain the following independent variables: Time (Month 8, 14, 20, 26, 32, 38), and survey covariates (JPO age, sex, and race/ethnicity, time in current position, highest/type degree, number of previous positions, salary, caseload size, job (dis)satisfaction rating, turnover intentions, and burnout rating). The Time effect will be tested to determine whether implementation outcomes change over time. The Covariate-by-Time interaction will be tested to determine whether the implementation outcomes changed differently over time for particular covariate values. Alternative time covariance structures (e.g., autoregressive) will be considered. Alternative models will be compared with Akaike Information Criterion (AIC), with lowest AIC indicating the better model statistically.

Step 2. The analyses will be similar to that described above in Step 1 except the implementation variables (readiness, climate support, leadership support, and collaborative alliance) will be used as independent variables to predict the dependent variable of implementation fidelity. Fidelity will be measured (in three separate models) using the SIC Pre-implementation score, the SIC Implementation score, and the SIC Sustainability score. In exploratory analyses, two addition fidelity dependent variables will be analyzed: percentage of activities performed, and Duration (number of days between activities). The analysis for Aim 4 describes how implementation climate and fidelity measures will be used in models as predictors of the SU cascade of care and opioid related outcomes.

Aim 3 Power. We require a total sample size of 384 enrolled (we will be conservative and enroll 395) JJ and CMHC staff for the implementation surveys. The actual analysis will employ linear mixed models that incorporate all staff participants with available implementation outcomes variables collected at multiple survey time points (Study Month 8, 14, 20, 26, 32, 38). The analysis models will actually also account for a second clustering variable (i.e., within each county, we will have surveys from staff of one

JJ system, and surveys from staff of one CMHC system), yielding 16 total clusters (8 counties by two JJ/CMHC sites within each county). However, the sample size inflation factor is greater with fewer clusters; therefore, for power calculations, we conservatively assumed 8 clusters instead of 16 total clusters. We used the PASS software to calculate power for a test comparing two means (control condition mean vs treatment condition mean) in a cluster-randomized step wedge design. Our step wedge design is an “incomplete design” in which a different number of clusters (i.e., counties) start the intervention at each step. Additional detail about the step wedge design, including clusters, cohorts, steps, timeline and design matrix, is provided below under the power section for Aim 4. Unlike Aim 4 which will have extensive (conservatively 6 months, actually 5 years) prior baseline administrative data for the control condition, the implementation scale data for Aim 3 is collected at a single baseline point (study month 8) and this will be used as the control condition measurement.

For the PASS input parameters, we assumed an intra-class (i.e., intra-cluster) correlation coefficient of 0.01, a two-sided test of mean difference (intervention vs control conditions), and an alpha of 0.05. We calculated power for 4 different hypothesized standardized mean differences (i.e., Cohen *d* effect size) consisting of 0.56, 0.60, 0.70, and 0.80. Results show that a total sample size of 384 survey respondents (i.e., average of 48 per cluster [*M* in step wedge design] with an average of 8 per cluster per 6-month time period [*m* in step wedge design]) will provide 80% to 98% power for detecting population mean differences that range from 0.56 to 0.80 SD units, for various continuous scores for the implementation outcomes (See PASS Report below). Thus, the planned total sample size of 395 survey respondents will provide slightly more than 80% power for an effect size of 1.56, which is slightly above the value of 1.50 which is considered a medium effect size according to Cohen’s criteria.

PASS Results Report for Power for Comparing Means for Aim 3

Design Type: Incomplete (Custom)
Test Statistic: Wald Z-Test
Hypotheses: H0: μ1 - μ2 = 0 vs. H1: μ1 - μ2 ≠ 0

Power	Design S/T/R	Clusters K	Cluster Size M/m	Sample Size N	Trt Mean μ1	Ctrl Mean μ2	Diff D1	Std Dev σ	ICC	Alpha
0.80	5/6/1	8	48/8	384	1.56	1.00	0.56	1.00	0.010	0.050
0.85	5/6/1	8	48/8	384	1.60	1.00	0.60	1.00	0.010	0.050
0.94	5/6/1	8	48/8	384	1.70	1.00	0.70	1.00	0.010	0.050
0.98	5/6/1	8	48/8	384	1.80	1.00	0.80	1.00	0.010	0.050

Report Definitions
S is the number of steps in the study design. S = T - 1.
T is the number of time periods in the study, including the baseline. T = S + 1
R is the number of times that each row of the custom design pattern matrix is replicated.
K is the total number of clusters to be randomized.
M is the average number of subjects per cluster.
m is the average number of subjects per cluster per time period.
N is total sample size from all clusters and time periods combined.
μ1 is the treatment mean, assuming the alternative hypothesis.
μ2 is the control, standard, reference, or baseline mean.
D1 = μ1 - μ2 is the difference assuming the alternative hypothesis (H1).
σ is the subject-to-subject standard deviation.
ICC is the intra-cluster correlation coefficient.

Summary Statements
A sample of 8 clusters in an incomplete (or custom) stepped-wedge cluster-randomized design with 6 time periods (including the baseline), 5 steps, and an average of 48 subjects per cluster with an average of 8 subjects per cluster per time period (for a total sample size of 384 subjects) achieves 80% power to detect a difference between means of 0.56. The treatment mean is assumed to be 1.56 under the alternative hypothesis. The control mean is 1.00. The

standard deviation is 1.00. The test statistic used is the two-sided Wald Z-Test. The ICC is 0.010, and the significance level of the test is 0.050.

References for PASS Report

Hussey, M.A., and Hughes, J.P. 2007. 'Design and analysis of stepped wedge cluster randomized trials'. Contemporary Clinical Trials, Volume 28, pages 182-191.

Hemming, K., and Girling, A. 2014. 'A menu-driven facility for power and detectable-difference calculations in stepped-wedge cluster-randomized trials'. The Stata Journal, Volume 14, pages 363-380.

Hemming, K., Lilford, R., and Girling A.J. 2015. 'Stepped-wedge cluster randomised controlled trials: a generic framework including parallel and multiple-level designs'. Statistics in Medicine, Volume 34, pages 181-196.

Baio G., et al. 2015. 'Sample size calculation for a stepped wedge trial'. Trials, 16: 354.

Hemming, K., and Taljaard, M. 2016. 'Sample size calculations for stepped wedge and cluster randomised trials: a unified approach'. Journal of Clinical Epidemiology, Volume 69, pages 137-146.

End of PASS Report

Aim 4 Comparative Effectiveness Analyses

Aim 4 Analyses

We will use standard step wedge analytic methods (see citations in reference list for PASS report). We will use repeated measures Poisson models to compare control vs LHS intervention conditions on the response rate ratio for the SU cascade outcomes and the opioid related outcomes. The Poisson or zero-inflated Poisson regression models will be performed using SAS GENMOD to fit repeated measures generalized linear models (GLIM) with generalized estimating equations (GEE) to account for the fact that event data for youth will be measured repeatedly over time and to account for the correlation due to within-organization clustering. The outcome data will be entered into the model as event counts for every 6-month period of the 36 months of implementation (6 months of control condition for all clusters, then 30 months of intervention), reported separately for each arrest record. Multiple arrests for the same youth will be used in the model. In the power analysis (described below), we conservatively based the power calculations on the number of unique youth arrested. The actual analysis will use a random effect to incorporate data from youth who have multiple arrests.

The amount of time (in days) exposed to the implementation for each adolescent arrestee will be accounted for in the rate ratio by defining the “offset” variable as the natural log of the number of days of exposure for every 6-month period of implementation. The primary denominator will be the number of arrested youth in the rolling intake cohort (e.g., % referred as a percentage of the number in the arrestee intake cohort). For some outcomes, another separate outcome will be derived and analyzed using a smaller denominator (e.g., % referred as a percentage of YJJ in need of treatment). The denominator for % in need (i.e., % screened positive) will be YJJ screened. The denominator for the % engaged will be the YJJ initiated. Because some youth will have multiple arrests, they could also have multiple screenings. In addition, for a single arrest and single screening, there could be multiple referral events over any 6-month period; for a single referral, there could be multiple treatment initiation events; and for a single treatment initiation, there could be multiple occurrences of an engaged event (i.e., 2+ behavioral health visits within 30 days after initiation) over any 6-month period. Therefore, the model will capture these multiple events as counts for every 6-month period.

Covariate-by-6-Month-Period interactions will be tested to determine whether the outcome response rates changed differently over time for different values of covariates.

In addition, youth-level covariates (demographics and health visit data) will also be included as predictors.

In the next step of the model, we will include organizational-level (i.e., county-by-system [JJ and CMHC]) survey predictors including staff characteristics (JPO age, sex, and race/ethnicity, time in current position, highest/type degree, number of previous positions, salary, caseload size, job (dis)satisfaction rating, turnover intentions, and burnout rating), implementation variables (readiness,

climate support, leadership support, and collaborative alliance), and fidelity variables (SIC Pre-implementation, Implementation score, and Sustainability scores; percentage of activities performed; and number of days between activities) to determine whether these survey variables predict the response rates for the SU cascade and opioid related outcomes. The implementation and fidelity measures will be entered as time-varying predictors because they will be available from multiple survey measurement time points as described above for the analysis of Aim 3.

Aim 4 Power

Based on available data, we anticipate 2,170 adolescents will be arrested per year from the 8 counties. The county-specific (i.e., cluster-specific) estimated annual arrests/referrals is shown below.

County	#referrals	year #referrals was reported
1	325	2017
2	190	2017
3	290	2017
4	328	2017
5	164	2017
6	706	2017
7	40	2018
8	127	2018
Total per year	2170	

However, we acknowledge some youth will be placed out of the home (DOC and residential placement). Additionally, some youth will migrate out of the community (which will be assessed by address data from state-wide databases; INPC, Medicaid, & DCS). Thus, we conservatively estimate that roughly 70% of 2,170 (i.e., 1,519) adolescent arrestees per year will be available in-home in the community from the 8 counties (4,557 total over 36 months of implementation which includes 30 months of intervention plus 6 prior months in which all cohorts experience the control condition under the baseline period). Based on an annual 30% recidivism rate, we estimate that 30% (1,367) of the projected 4,557 arrests for this trial will represent repeated arrests from the same youth, yielding an estimated 3,190 unique youth available in the administrative data over the 36 months of the primary outcome measurement period. We will actually collect and use administrative data for 5 years (not just 6 months) prior to the start of intervention; however, to be conservative, the step wedge power calculation assumed only 6 months of prior baseline administrative data (i.e., 36 months of primary administrative measurement). Additionally, although all data (including multiple arrests for the same youth) will be used in the model, to be conservative for the power analysis, we assumed that the maximum sample size available for the power calculations is the number of unique youth (3,190).

Our design has 8 counties (i.e., clusters). A projected maximum available sample size of 3,190 unique youth, over the 36-month primary evaluation period, yields: (1) 531 youth per 6-month time period, (2) an average of 398 maximum youth per cluster over 36 months (*M* in the Step Wedge Design), and (3) an average of 66 youth per cluster per 6-month time period (*m* in Step Wedge design).

We used the PASS software to calculate power for a test of two poisson rates (control condition event rate vs treatment condition event rate) in a cluster-randomized step wedge design. Our step wedge design is an “incomplete design” in which a different number of clusters (i.e., counties) start the intervention at each step. Given 8 clusters and practical considerations, we decided to initiate intervention for 3 clusters in cohort 1, then in the next step another 3 clusters in cohort 2, and then in the next step another 2 clusters in cohort 3.

We input the following customized design matrix into the PASS software. The timeline, shown elsewhere in this application, is displayed in 3-month increments to show specific details such as the 3 months of dissemination at the end of study. For the step wedge design matrix, each time period

represents a 6-month time interval because the administrative outcome data will be measured every 6 months, and the cohorts will also be started on intervention in 6-month steps of our step wedge design.

Step Wedge Design Matrix (Incomplete Design: K = 8 Clusters, S = 5 Steps, T = 6 Times Periods)

		Time (6-month intervals)					
		1	2	3	4	5	6
		Intervention Months					
Cohort	County Cluster	-6 to 0	1 to 6	7 to 12	13 to 18	19 to 24	25 to 30
1	1	0	1	1	1	1	1
1	2	0	1	1	1	1	1
1	3	0	1	1	1	1	1
2	4	0	0	1	1	1	1
2	5	0	0	1	1	1	1
2	6	0	0	1	1	1	1
3	7	0	0	0	1	1	1
3	8	0	0	0	1	1	1

For the PASS input parameters, we assumed an intra-class (i.e., intra-cluster) correlation coefficient of 0.01, a two-sided test of the rate ratio (intervention vs control conditions), and an alpha of 0.05. We calculated power for 4 different hypothesized poisson rate ratios (effect size) ranging from 1.25 to 1.40 by .05. Results show that a total sample size of 2,400 youth (i.e., average of 300 unique youth per cluster with an average of 50 youth per cluster per 6-month time period) will provide 80% to 99% power for detecting population rate ratios, for various outcomes derived from administrative data described elsewhere, ranging from 1.25 to 1.40 (See PASS Report below). Thus, the available total sample size of 3,190 unique youth (i.e., average of 398 unique youth per cluster with an average of 66 youth per cluster per 6-month time period) will provide more than ample power (e.g., exceeding 80% power for a rate ratio of 1.25). In addition, the actual analysis will include youth with multiple arrests which will increase power further.

PASS Results Report for Power for Comparing Poisson Rates for Aim 4

Design Type: Incomplete (Custom)
Test Statistic: Wald Z-Test
Hypotheses: H0: $\lambda1/\lambda2 = 1$ vs. H1: $\lambda1/\lambda2 \neq 1$

Power	Design S/T/R	Clusters K	Cluster Size M/m	Sample Size N	Trt Rate λ1	Ctrl Rate λ2	Ratio RR1	ICC	Alpha
0.80	5/6/1	8	300/50	2400	1.25	1.00	1.25	0.010	0.050
0.91	5/6/1	8	300/50	2400	1.30	1.00	1.30	0.010	0.050
0.97	5/6/1	8	300/50	2400	1.35	1.00	1.35	0.010	0.050
0.99	5/6/1	8	300/50	2400	1.40	1.00	1.40	0.010	0.050

Report Definitions
S is the number of steps in the study design. S = T - 1.
T is the number of time periods in the study, including the baseline. T = S + 1
R is the number of times that each row of the custom design pattern matrix is replicated.
K is the total number of clusters to be randomized.
M is the average number of subjects per cluster.

m is the average number of subjects per cluster per time period.
N is total sample size from all clusters and time periods combined.
$\lambda 1$ is the average treatment event rate per unit time, assuming the alternative hypothesis.
$\lambda 2$ is the average control, standard, reference, or baseline event rate per unit time.
RR1 = $\lambda 1/\lambda 2$ is the rate ratio assuming the alternative hypothesis (H1).
ICC is the intra-cluster correlation coefficient.

Summary Statements

A sample of 8 clusters in an incomplete (or custom) stepped-wedge cluster-randomized design with 6 time periods (including the baseline), 5 steps, and an average of 300 subjects per cluster with an average of 50 subjects per cluster per time period (for a total sample size of 2400 subjects) achieves 80% power to detect a ratio of 1.25. The treatment event rate is assumed to be 1.25 under the alternative hypothesis. The control event rate is 1.00. The test statistic used is the two-sided Wald Z-Test. The ICC is 0.010, and the significance level of the test is 0.050.

References for PASS Report

Hussey, M.A., and Hughes, J.P. 2007. 'Design and analysis of stepped wedge cluster randomized trials'. Contemporary Clinical Trials, Volume 28, pages 182-191.

Hemming, K., and Girling, A. 2014. 'A menu-driven facility for power and detectable-difference calculations in stepped-wedge cluster-randomized trials'. The Stata Journal, Volume 14, pages 363-380.

Hemming, K., Lilford, R., and Girling A.J. 2015. 'Stepped-wedge cluster randomised controlled trials: a generic framework including parallel and multiple-level designs'. Statistics in Medicine, Volume 34, pages 181-196.

Baio G., et al. 2015. 'Sample size calculation for a stepped wedge trial'. Trials, 16: 354.

Hemming, K., and Taljaard, M. 2016. 'Sample size calculations for stepped wedge and cluster randomised trials: a unified approach'. Journal of Clinical Epidemiology, Volume 69, pages 137-146.

End of PASS Report

The PASS software performs step wedge power calculations for the “cross-sectional” step wedge design which assumes that different youth are measured within each cluster at each time point. Our design is primarily a cohort (i.e., repeated measures) design in which youth are measured repeatedly at each point in time; however, administrative data for some youth will become unavailable at later points in time due to moving out of the system, therefore, our design will contain both repeated measures and cross-sectional data. The PASS cross-sectional design assumption is conservative for calculating power for our design because the test of intervention vs control conditions will be more powerful when the same (instead of different) youth are measured repeatedly.

Missing Data

For analyses of all aims, bias due to missing data will be handled by using the state-of-the-art multiple imputation method with 100 multiply include data sets using the SAS MI and MIANALYZE procedures.
