# Supplementary material for: Learning Health Systems and Substance Use Care Cascade Achievement Among Justice-Involved Youth: A Cluster-Randomized Stepped-Wedge Clinical Trial
Source: JAMA Netw Open. 2026 Feb 10;9(2):e2558222. doi: 10.1001/jamanetworkopen.2025.58222 (PMC12892156; doi:10.1001/jamanetworkopen.2025.58222)
Supplement: Supplement 2. — eTable 1. Mean Number of Days Between Arrest and Cascade Step Achieved by Study Phase eTable 2. Exploring the Effect of the LHS on Time From Arrest to Treatment Initiation and Engagement eTable 3. Sensitivity Analysis With Bonferroni Adjustment [file jamanetwopen-e2558222-s002.pdf]

Supplementary Online Content

Aalsma MC, Schwartz K, Sun D, et al. Learning health systems and substance use care cascade achievement among justice-involved youth. *JAMA Netw Open.* 2026;9(2):e2558222. doi:10.1001/jamanetworkopen.2025.58222

eTable 1. Mean Number of Days Between Arrest and Cascade Step Achieved by Study Phase

eTable 2. Exploring the Effect of the LHS on Time From Arrest to Treatment Initiation and Engagement

eTable 3. Sensitivity Analysis With Bonferroni Adjustment

This supplementary material has been provided by the authors to give readers additional information about their work.

© 2026 Aalsma MC et al. *JAMA Network Open*.

eTable 1. Mean Number of Days Between Arrest and Cascade Step Achieved by Study Phase

	Pre-Intervention Control	LHS Intervention Implementation
Arrested to Screened		
N	1,972	2,145
Mean (SD)	161 (291)	76 (142)
Median (Q1,Q3)	43 (14, 146)	27 (7, 68)
Arrested to Referred		
N	1,768	1,717
Mean (SD)	203 (327)	124 (184)
Median (Q1,Q3)	69 (20, 203)	56 (12, 148)
Arrested to Initiated		
N	1,337	1,302
Mean (SD)	312 (426)	158 (227)
Median (Q1,Q3)	125 (16, 447)	58 (8, 216)
Arrested to Engaged		
N	856	825
Mean (SD)	244 (393)	122 (195)
Median (Q1,Q3)	69 (17, 273)	39 (12, 146)

© 2026 Aalsma MC et al. *JAMA Network Open*.

eTable 2. Exploring the Effect of the LHS on Time From Arrest to Treatment Initiation and Engagement

As reported in manuscript Table 2, the "main effect" of LHS intervention was significantly associated with lower probability of (i.e., longer time) from youth first arrest to treatment initiation (HR=0.68, 95% CI=0.53-0.84, p<0.01) and from arrest to treatment engagement (HR=0.64, 95% CI=0.49-0.79, p<0.01). However, the interaction of treatment effect and arrest time (i.e., time from study start to arrest time) was also significant for the transitions from arrested to initiated and arrested to engaged in treatment, implying arrest time moderates the effect of the intervention on treatment initiation and engagement. Further exploration of these interaction results indicated that compared to pre-intervention, the LHS was associated with reduced duration between arrest and treatment initiation and engagement, but only during the later part of the study; specifically, this LHS effect began approximately 3.5-4 years after the start of the observation period through end of study, which roughly coincides with the COVID-19 pandemic subsiding (i.e., 2022-2023). The conclusion of 3.5-4 years as the breakeven point after which the effect switched from being better for control to being better for LHS came from the raw coefficient results (log hazard ratio) as reported in the table below. With the interaction of time, the overall effect of the intervention is (coef(Treatment) + coef(Treatment*Time). In this data, the unit of time since arrest is year. So, for example, the breakeven point for treatment engagement is 0.445/0.126= 3.53 year, and the breakeven point for treatment initiation is 0.384/0.094 = 4.09 year. When the arrest time is shorter (i.e., earlier in the study period) than the breakeven point, the coefficient is negative (i.e., HR < 1) and duration between steps is longer. After the 3.5-4 years, HR > 1, indicating shorter duration.

Outcome: Time from arrest to cascade step; Results presented as log hazard ratios

	Screened (95% CI)	Referred (95% CI)	Initiated (95% CI)	Engaged (95% CI)
Treatment (LHS intervention implementation)	0.514*** (0.182, 0.845)	0.387 (-0.175, 0.949)	-0.384*** (-0.613, -0.155)	-0.445*** (-0.675, -0.215)
Arrest Time	-0.009 (-0.124, 0.106)	-0.037 (-0.143, 0.069)	0.016 (-0.077, 0.110)	-0.047 (-0.172, 0.078)
Treatment x Arrest Time	-0.093 (-0.276, 0.091)	-0.087 (-0.239, 0.065)	0.094** (0.007, 0.181)	0.126*** (0.041, 0.211)
Male vs Female	-0.038 (-0.132, 0.056)	-0.135** (-0.253, -0.017)	-0.192*** (-0.293, -0.091)	-0.216*** (-0.296, -0.137)
White vs Other Races	0.190*** (0.085, 0.296)	0.405*** (0.278, 0.532)	0.444*** (0.255, 0.633)	0.390*** (0.158, 0.623)
Age at the First Arrest	-0.065*** (-0.093, -0.036)	-0.105*** (-0.135, -0.074)	-0.154*** (-0.187, -0.121)	-0.162*** (-0.198, -0.126)

**p*<0.10; ***p*<0.05; ****p*<0.01*

© 2026 Aalsma MC et al. *JAMA Network Open*.

eTable 3. Sensitivity Analysis With Bonferroni Adjustment

As reported in manuscript Table 2, the Benjamini-Hochberg (BH) adjustment was made to the time to event analysis to control the false discovery rate (FDR) across the 24 pre-specified comparisons. BH was selected rather than the more conservative Bonferroni correction because BH provides greater power while still maintaining rigorous control over the expected proportion of false discoveries, particularly when analyzing correlated outcomes. For transparency, a sensitivity analysis was conducted using the Bonferroni adjustment. As expected, this approach yielded more conservative results than the BH adjustment.

Summary of time to event analysis results with Bonferroni adjustment

	(time from first arrest to cascade step)			
	Screened<br>HR (95% CI)	Referred<br>HR (95% CI)	Initiated<br>HR (95% CI)	Engaged<br>HR (95% CI)
Treatment<br>(ADAPT Implementation)	**1.67***<br>(1.12, 2.23)	1.47<br>(0.65, 2.30)	**0.68****<br>(0.53, 0.84)	**0.64*****<br>(0.49, 0.79)
Arrest Time	0.99<br>(0.88, 1.12)	0.96<br>(0.86, 1.07)	1.02<br>(0.92, 1.11)	0.95<br>(0.83, 1.07)
Treatment x Arrest Time	0.91<br>(0.74, 1.08)	0.92<br>(0.89, 0.95)	1.10<br>(1.00, 1.20)	**1.13***<br>(1.04, 1.23)
Male vs Female	0.96<br>(0.87, 1.05)	0.87<br>(0.77, 0.98)	**0.83*****<br>(0.74, 0.91)	**0.81*****<br>(0.74, 0.87)
White vs Other Races	**1.21*****<br>(1.08, 1.34)	**1.50*****<br>(1.31, 1.69)	**1.56*****<br>(1.26, 1.85)	**1.48****<br>(1.13, 1.82)
Age at First Arrest	**0.94*****<br>(0.91, 0.97)	**0.90*****<br>(0.87, 0.93)	**0.86*****<br>(0.83, 0.89)	**0.85*****<br>(0.82, 0.88)

**p<0.1; ** p<0.05; ***p<0.01*

© 2026 Aalsma MC et al. *JAMA Network Open*.
