# Supplementary material for: Learning Health Systems and Substance Use Care Cascade Achievement Among Justice-Involved Youth: A Cluster-Randomized Stepped-Wedge Clinical Trial
Source: JAMA Netw Open. 2026 Feb 10;9(2):e2558222. doi: 10.1001/jamanetworkopen.2025.58222 (PMC12892156; doi:10.1001/jamanetworkopen.2025.58222)
Supplement: Supplement 3. — Data Sharing Statement [file jamanetwopen-e2558222-s003.pdf]

Data Sharing Statement

Aalsma. Learning Health Systems and Substance Use Care Cascade Achievement Among Justice-Involved Youth. *JAMA Netw Open*. Published February 10, 2026. doi:10.1001/jamanetworkopen.2025.58222

Data
Additional Information: Clinicaltrials.gov; NCT04499079
Data available: No

Additional Information
Explanation for why data not available: Existing data use agreements between the authors' university and the state Supreme Court do not allow sharing youth legal system records unless at an aggregate level.
